# Reovirus genomic diversity confers plasticity for protease utility during adaptation to intracellular uncoating

**DOI:** 10.1128/jvi.00828-23

**Published:** 2023-09-25

**Authors:** Qi Feng Lin, Casey X. L. Wong, Heather E. Eaton, Xiaoli Pang, Maya Shmulevitz

**Affiliations:** 1 Department of Medical Microbiology and Immunology, Li Ka Shing Institute of Virology, University of Alberta, Edmonton, Alberta, Canada; 2 Department of Laboratory Medicine and Pathology, University of Alberta, Edmonton, Alberta, Canada; 3 Public Health Laboratories (ProvLab), Alberta Precision Laboratories (APL), Edmonton, Alberta, Canada; St. Jude Children's Research Hospital, Memphis, Tennessee, USA

**Keywords:** reovirus, adaptation, protease, host-virus interaction, virus entry, genetic diversity

## Abstract

Mammalian orthoreoviruses (reoviruses) infect many mammals and provide a safe model to study enteric viruses. Knowledge of reoviruses is derived from prototypic lab strains, notably T1L and T3D. Based on these prototypic strains, reoviruses can efficiently utilize gut or lysosomal proteases for uncoating and penetration into host cytoplasm. To discover natural reovirus characteristics, we isolated four genetically diverse wastewater reovirus samples and compared their genetic and phenotypic characteristics to those of T1L/T3D. On L929 cells, the most commonly used model for reovirus studies, naturally derived reoviruses displayed lower fitness than T1L/T3D, evident from smaller plaques and higher relative particle-to-PFU ratios. In contrast to T1L/T3D, which are uncoated efficiently by lysosomal-mediated proteolysis, naturally derived reoviruses displayed poor uncoating kinetics. Exposure of naturally derived reoviruses to gut proteases led to rapid extracellular uncoating and significantly improved infectivity relative to T1L/T3D, indicating that wild reoviruses depended more on extracellular gut proteases. To discover if reovirus can adapt to new host proteases, naturally derived samples were passaged on L929 cells, and large and small plaque-forming isolates were evaluated. Larger plaque sizes correlate with faster intracellular uncoating. Whole-genome sequencing identified the outer capsid σ3 protein as the determinant of uncoating proficiency between wild reoviruses. Interestingly, mutations that permitted intracellular uncoating were found in 0.01% of the parental quasispecies, suggesting that variants with altered protease sensitivity were rapidly selected for during cell culture propagation. Altogether, these findings indicate that naturally circulating reoviruses are more reliant on gut proteases, but the genetic diversity of reovirus quasispecies bestows tremendous plasticity toward protease utility.

**IMPORTANCE:**

Reoviruses infect many mammals and are widely studied as a model system for enteric viruses. However, most of our reovirus knowledge comes from laboratory strains maintained on immortalized L929 cells. Herein, we asked whether naturally circulating reoviruses possess the same genetic and phenotypic characteristics as laboratory strains. Naturally circulating reoviruses obtained from sewage were extremely diverse genetically. Moreover, sewage reoviruses exhibited poor fitness on L929 cells and relied heavily on gut proteases for viral uncoating and productive infection compared to laboratory strains. We then examined how naturally circulating reoviruses might adapt to cell culture conditions. Within three passages, virus isolates from the parental sewage population were selected, displaying improved fitness and intracellular uncoating in L929 cells. Remarkably, selected progeny clones were present at 0.01% of the parental population. Altogether, using reovirus as a model, our study demonstrates how the high genetic diversity of naturally circulating viruses results in rapid adaptation to new environments.

## INTRODUCTION

Viruses are obligate intracellular parasites that have co-evolved with living organisms for millions of years (1). Consequently, each virus species has adapted to infect and hijack the cellular machinery of its natural hosts, whether they be bacteria, plants, or specific cells within animals. The reliance on specific cellular factors available in distinct hosts has been characterized for some viruses (2 – 4). For example, the preference of avian compared to human strains of influenza A virus for sialic acids with slightly different molecular structures contributes to the host tropism of these strains (3). Additionally, different cell types within a species can express distinct molecular factors essential for some viruses (5 – 9). The liver-specific microRNA-122, for instance, is essential for hepatitis C virus replication in the liver (9). Despite the long history of viral adaptation, researchers often study viruses using simplified model systems that lack the complexity and diversity found in natural viral infections (7, 10 – 12). By characterizing molecular features that govern viral replication in new environments, we can better understand how viruses adapt to new hosts or to distinct niches within a given host. Such knowledge could help appreciate virus progression across and within domains of life and could, for example, help predict virus adaptation to new pressures.

Viruses that infect animals through the gastrointestinal tract must be, especially, well adapted to withstand harsh digestive conditions, such as pH changes and proteolytic enzymes (13 – 15). To achieve successful enteric infection, viruses such as rotavirus, mammalian orthoreovirus, and human astrovirus have evolved hyper-stable capsid structures and rely on proteolytic processing by intestinal enzymes to activate viral particles for infection (5, 12, 16 – 20). Culturing these enteric viruses in a laboratory without intestinal factors can be challenging; trypsin activates viral particles through proteolysis and is often added to culturing media when growing rotavirus and human astrovirus for experiments (12, 16). Furthermore, clinical rotavirus samples can sometimes require several rounds of passaging in primary cells before being able to infect continuous cell lines efficiently (11, 12). Thus, some enteric viruses are maladapted to replicate in commonly used cell culture systems, which can impose different selective pressures compared to the natural niche in the intestine. Mammalian orthoreovirus (reovirus) is an interesting enteric virus that similarly benefits from activation by digestive enzymes during intestinal infection yet has been successfully propagated and studied in non-enteric culture systems. In fact, a laboratory strain of reovirus (T3D^PL^) that was propagated on tumorigenic cells displays high fitness in numerous cancer cell cultures and is now undergoing human clinical trials as an oncolytic agent for many non-enteric cancers (21 – 23). In nature, reoviruses can infect virtually all mammals through the gastrointestinal tract without causing disease in immunocompetent hosts (24, 25). Reovirus is also well adapted to persist in the natural environment, given that it is highly abundant in natural water sources as well as in untreated and treated wastewaters (13). The apparent versatility of reoviruses makes them a useful model to study factors required for optimal viral replication in both intestinal and non-intestinal environments.

Reoviruses of serotypes 1, 2, and 3 were isolated in the 1950s, and prototypic strains of each serotype (T1L, T2J, and T3D) have been propagated in transformed cell cultures, typically in the tumorigenic L929 mouse fibroblast cell line, for research purposes. Over 70 years of studies on these prototypic strains in culture and animal models have revealed fundamental aspects of reovirus infection. Cell attachment for all three prototypic reoviruses utilizes the viral σ1 proteins to bind sialic acids and junctional adhesion molecules on host cell surfaces (26 – 30). However, T1L and T3D were found to exhibit distinct sialic acid linkage preferences via different domains on their σ1 proteins (28 – 30). The σ1 protein also determines susceptibility to antibody neutralization and hence dictates serotype designation (31). Reoviruses are composed of two capsids: a core capsid maintained during replication and an outer capsid that protects the cores and must be removed for infection to progress. When exposed to the digestive enzymes trypsin or chymotrypsin extracellularly, or to lysosomal proteases (mainly cathepsins B and L) following receptor-mediated endocytosis, prototypic reoviruses undergo proteolytic processing/uncoating of the outer capsid (32 – 35). Specifically, infectious subviral particles (ISVPs) are produced by complete degradation of the outermost σ3 capsid protein and cleavage of the underlying μ1 protein into δ and φ fragments, which are involved in membrane penetration (34, 35). Penetration of ISVPs results in transcriptionally active core particles in the cytoplasm that establish productive infection (34, 36, 37). Despite a generalized understanding of the processes involved in reovirus replication, it is yet to be known whether these processes differ under distinct host and environmental factors and for distinct naturally derived reoviruses.

Subtle differences among prototypic reovirus strains have already been characterized. For example, T1L and T3D exhibit strain-specific differences in their efficiency for membrane penetration, apoptosis induction, and capacity to infect *in vivo* through the intestinal route (34, 38 – 40). Even the same 1950 prototypic reovirus isolates propagated in different laboratories (e.g., T3D^PL^ vs T3D) now exhibit distinct features in their replication kinetics, cytokine induction profiles, and oncolytic activities (21, 41, 42). Moreover, the cell entry mechanism and innate immune response vary depending on whether the infection is initiated by virions or ISVPs of the same reovirus strain (43, 44). The behavioral diversity among prototypic strains raises questions about which aspects of reovirus infection are shared with naturally derived viruses versus those resulting from culture adaptation. Furthermore, by testing if specific infection processes show preference for specific host and niche factors, we could reveal the basic dynamics of virus fitness and adaptation.

In this study, naturally circulating reoviruses from the Edmonton municipal wastewater (effluent) were characterized genetically and molecularly using the L929 cell culture system used to study prototypic strains. Whole-genome sequencing revealed that reoviruses in nature exhibit high genetic diversity distinct from prototypic T1L and T3D laboratory strains. Naturally derived reoviruses produced smaller plaques and had debilitated intracellular uncoating in L929 cells relative to lab-adapted strains, despite similar attachment to cells. Importantly, pre-treatment with intestinal lavage increased the infectivity of effluent isolates toward L929 cells significantly more than T1L and T3D, suggesting that naturally derived reoviruses depend more on extracellular intestinal factors for infection. *In vitro* digestion experiments revealed that naturally circulating reoviruses can vary in their susceptibility to proteolysis by intestinal enzymes from different source species. In just three passages on L929 cells, progeny viruses emerged that produced larger plaques and exhibited enhanced cathepsin-mediated uncoating relative to the parental effluent quasispecies. Increased sensitivity to intracellular uncoating was correlated with polymorphisms in the outer capsid σ3 proteins that were present at very low frequency in the parental quasispecies. Overall, our findings demonstrate that the majority of naturally circulating reovirus particles rely on intestinal proteases from specific species for uncoating and infection. However, the existence of low-frequency mutations among the natural quasispecies likely enables rapid adaptation to intracellular proteases under selective pressure.

## RESULTS

### Naturally circulating wastewater reovirus isolates are genetically distinct from prototypic strains, other known field isolates, and each other

Given that most of our knowledge about reoviruses comes from studying prototypic strains cultured in laboratories for decades, we sought to determine if naturally circulating reoviruses behaved like lab-adapted prototypes in cell culture systems. To obtain naturally circulating reovirus isolates, primary effluent samples from the city of Edmonton were screened by RT-qPCR for reovirus S1 mRNAs. Four S1 mRNA-positive samples were then validated to contain reovirus by plaque titration and immunohistochemistry using polyclonal antibodies (anti-reovirus) that recognize reovirus outer capsid proteins σ3 and μ1C in a serotype-independent manner. The goal was to study these naturally circulating reoviruses with minimal passage; however, in order to obtain enough virus for experiments, primary effluent samples were passaged twice without clonal selection of single plaques to maintain the original quasispecies as much as possible to produce “parental” effluent isolates. Total reovirus particles were purified using the Capto Core 700 in-slurry method described previously (45), and the genomes of the four naturally derived reovirus samples were evaluated by whole-genome sequencing to establish how similar or diverse they were from other reovirus strains reported in the literature as well as from each other. Illumina MiSeq reads were assembled *de novo* using Shovill SPAdes Version 1.1.0 on Galaxy.org to produce contigs that were annotated with NCBI-BLASTN (46, 47). For each sewage reovirus sample, contigs corresponding to the S1, S2, S3, S4, M1, M2, M3, L1, L2, and L3 genome segments were achieved (Table S1).

**FIG 1:**
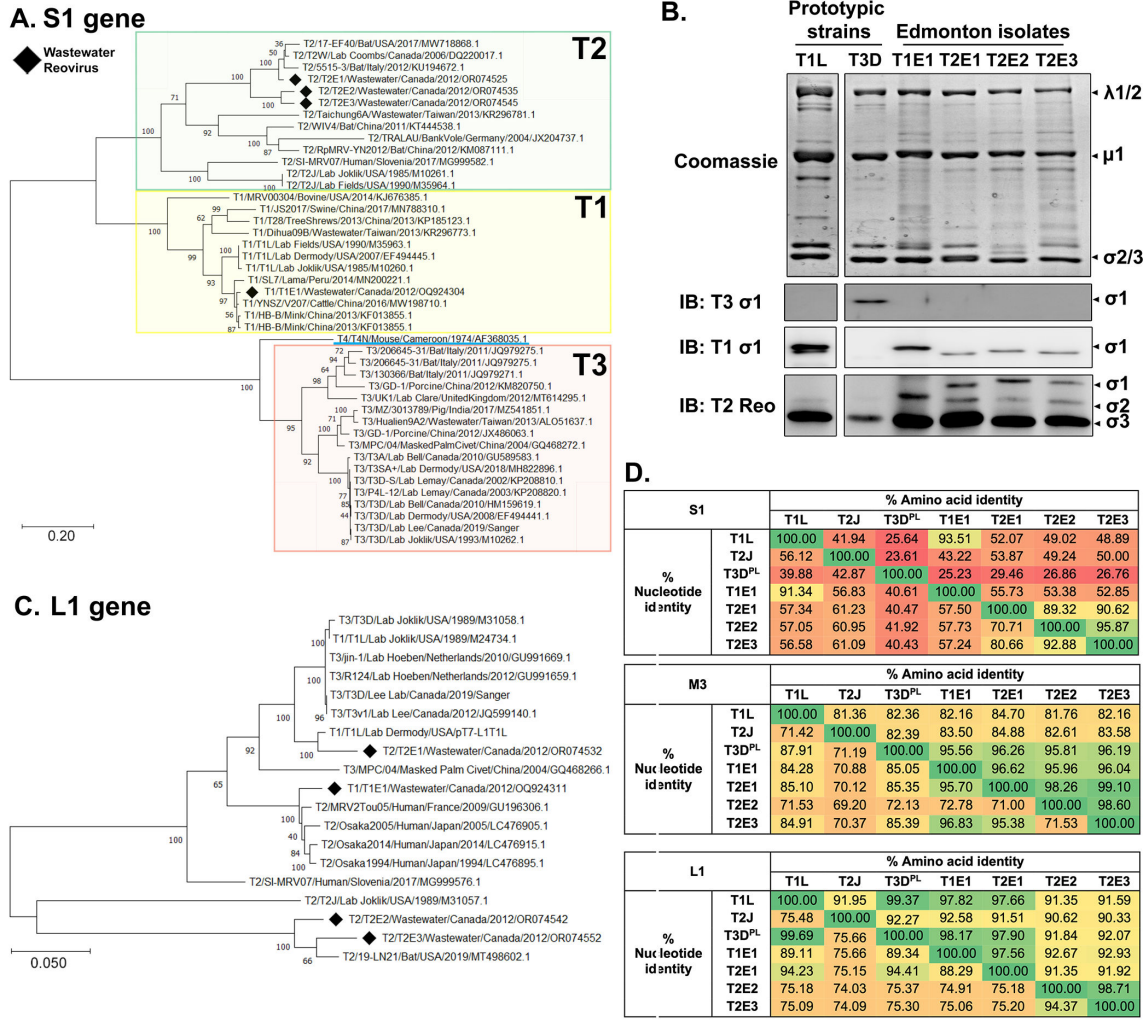
Natural reovirus isolates are genetically diverse and distinct from both prototype strains and other known field isolates. (**A–D**) Whole-genome sequencing was performed on genomic RNA extracted from purified effluent reovirus particles. (**A**) The S1 gene nucleotide sequence of naturally acquired isolates (black diamond) and others available on the NCBI GenBank was aligned using ClustalOmega (https://www.ebi.ac.uk/Tools/msa/clustalo/) with default settings. Phylogenetic trees were generated using the maximum-likelihood method based on the Tamura-Nei model with 1,000 bootstrap replicates on MEGA 11 software (48). Horizontal branch lengths are drawn to scale, with scale bars representing the number of substitutions per site. (**B**) Purified reoviruses were run on SDS-PAGE gel and either stained with Coomassie dye or probed by Western blot using serotype-specific polyclonal serum against T1, T2, or T3 σ1 protein/reovirus. (**C**) Similar to (**A**), except the phylogenetic tree was constructed based on the nucleotide sequence of the L1 polymerase gene. (**D**) Percent identity matrices comparing the S1, M3, or L1 genes of prototypic lab strains and Edmonton isolates at the nucleotide (*y*-axis) and amino acid levels (*x*-axis) following pairwise comparison analysis by ClustalOmega. Transition from red to green, representing an increasing percent identity.

Reoviruses are historically classified into serotypes based on cross-neutralization by polyclonal serum, for which the S1 gene-encoded σ1 cell attachment protein serves as the major determinant (31). The serotype designation of the naturally derived reovirus samples was therefore achieved by comparing S1 genome sequences using MEGA11 software (48). The S1 sequence of one of the sewage isolates was grouped with serotype 1 (T1) reoviruses and was accordingly named “T1E1” where E refers to Edmonton. The remaining three sewage reovirus samples were grouped with serotype 2 (T2) viruses and were accordingly named “T2E1,” “T2E2,” and “T2E3” (Fig. 1A). Both the BLAST and phylogenetic tree analyses indicated that the T1E1 S1 was most closely related to the YNSZ/V207/2016 isolate from cattle in China, with a nucleotide (nt) identity of 98.2% (Table S1). The S1 of T2E1 was most closely related to T2W identified in Canada (95.2% nt identity), while both T2E2 and T2E3 bore the closest relationship to the 17-EF40 isolate from USA bats with 82.2% nt identity. To further validate the relationships of the four sewage samples to prototypic reovirus serotypes, viruses were subjected to denaturing electrophoresis followed by Coomassie staining to visualize the major structural proteins λ1/2, μ1C, σ2, and σ3 and Western blot analysis to evaluate cross-reactivity to polyclonal rabbit antibodies generated specifically to T1L (T1 prototype), T2J (T2 prototype), or T3D (T3 prototype) σ1 proteins. The major structural proteins λ1/2, μ1, σ2, and σ3 of all viruses were visible at similar intensities by Coomassie staining. Conversely, only T3D σ1 was recognized by anti-T3D antibodies (Fig. 1B), consistent with the S1 sequence data described above. Moreover, while T1L and T1E1 σ1 proteins were strongly recognized by T1L-specific antibodies, T2E1, T2E2, and T2E3 σ1 proteins were recognized by anti-T1L serum but to a weaker extent; this is likely because T1 and T2 σ1 proteins are more closely related phylogenetically (Fig. 1A). Importantly, anti-T2J antibodies only recognized the σ1 proteins of T2E1, T2E2, and T2E3, supporting their genetic classification as T2 viruses. Altogether, there is strong support for the designation of T1E1 as serotype T1 and T2E1/T2E2/T2E3 as serotype T2.

Given that reovirus genomes are composed of 10 segments that can reassort, genetic analysis solely based on S1 overlooks the possibility for reassortment among other genes (40, 49). Since viruses are commonly classified based on the viral polymerase, a phylogenetic analysis of the reovirus λ3 polymerase-coding genome segment (L1) was conducted. Based on L1 sequences, the closest relative to T1E1 was now the serotype 2 human MRV2Tou05 isolate from France; T2E1 was now most closely related to the prototypic T1L virus, while T2E2 and T2E3 remained most closely related to the serotype 2 USA bat 17-EF40 isolate (Fig. 1C). The same phylogenetic analysis was performed on the non-structural gene, μNS-encoding M3 gene, which revealed yet another pattern of genetic relationship (Fig. S1A). These results suggest that extensive reassortments have occurred in nature and that the serotypes based on the S1 gene provide little relationship information for the remaining genes.

The analyses so far were based on nucleotide sequences, but high nucleotide divergence can still result in high amino acid conservation due to synonymous codons (49, 50). To assess virus protein sequence conservation, pairwise comparisons were made among the four sewage samples (T1E1, T2E1, T2E2, and T2E3) and the prototypic virus of each serotype (T1L, T2J, and T3D^PL^) to obtain percent identity for both nucleotides (*y*-axis) and amino acids (*x*-axis). Overall, relative nucleotide divergence was reflected in the amino acid sequences (Fig. 1D and S1B). Consistent with past literature, the S1 gene was the most divergent (51); for example, the T1E1 and T3D^PL^ S1 genes were only 40.61% and 25.23% identical at the nucleotide and amino acid levels, respectively. The other genes consistently had >70% nucleotide and >80%–90% amino acid identities, congruent with a recent study (52). Taken together, the four natural Edmonton reovirus isolates are genetically distinct from prototypic strains, any known reovirus isolates, and even from each other despite their geographic closeness.

### Naturally acquired reoviruses have poor overall virus fitness and intracellular uncoating in L929 cells

Prototypic T1L and T3D^PL^ reovirus lab strains have been passaged on L929 mouse fibroblast cells for decades and can infect L929 cells with high proficiency. These reovirus lab strains efficiently bind L929 cells and undergo endocytosis (28, 29, 33). Next, prototypic reoviruses undergo intracellular uncoating to produce transcriptionally active cores; specifically, lysosomal cathepsins mediate outer capsid σ3 protein degradation and cleavage of the underlying μ1C protein into a membrane-penetrating δ fragment (17, 34, 35). However, it remains to be explored whether natural reoviruses that do not undergo lab adaptation also efficiently perform all steps of infection in L929 cells or if they have unique characteristics that may have been lost during cell culture propagation.

**FIG 2:**
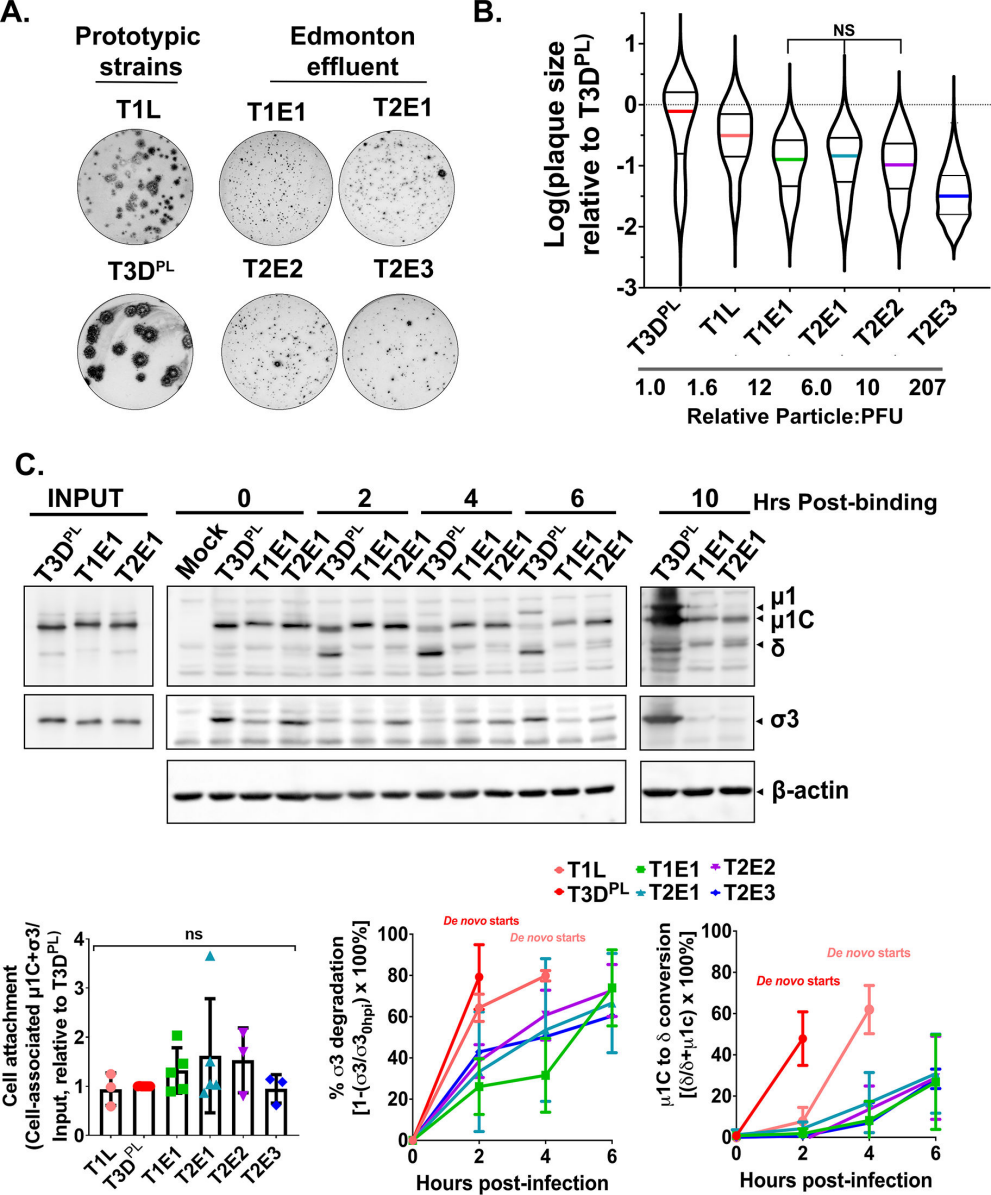
Naturally acquired reoviruses produce small plaques and have delayed intracellular uncoating in L929 cells. (**A**) Monolayers of L929 cells were infected with either T1L, T3D^PL^, T1E1, T2E1, T2E2, or T2E3 and overlaid with agar-containing media. Cells were fixed at 4 days post-infection prior to blocking and probing using rabbit polyclonal anti-reovirus as the primary antibody and an AP-conjugated secondary antibody. The cells were then processed for BCIP/NBT staining to generate plaques representing infected cells. (**B**) Foci size was quantified using ImageJ and plotted relative to T3D^PL^. Data are presented as a violin plot of log (relative plaque size) summarizing the data from *N* = 3 biological replicates. All pairwise comparisons are *P* < 0.0001 except those indicated as not significant (ns), which are *P* > 0.05. (**C**) L929 cells were synchronously infected with T1L, T3D^PL^, T1E1, T2E1, T2E2, or T2E3 at 4°C for 1 hour. Following incubation, cells were washed twice with serum-free media and placed in growth media at 37°C, representing 0 hpi. At the indicated timepoints, cells were harvested using RIPA buffer and processed for immunoblotting using anti-reovirus and anti-β-actin antibodies. The relative protein band intensities of cell-associated viral particles relative to the original inocula (Input) were used to calculate percent cell-bound particles at 0 hpi (bottom left). The percent uncoating was calculated as the relative σ3 protein band intensities relative to 0 hpi (bottom middle) or proportion of δ relative to δ + μ1C (bottom right). Error bars correspond to the standard deviation from at least *N* = 3 biological replicates. ns >0.05. All statistical analysis was done by one-way ANOVA with Tukey’s multiple comparison test.

To compare how well cell culture-adapted versus naturally derived reoviruses infect L929 cells, plaque assays were performed, and plaque size was used as an overarching measurement of how well the viruses bind, enter, replicate, and disseminate among L929 cells. Standard plaque titration was performed on L929 cells using serial dilutions of viruses, and the dilutions that gave similar numbers of plaques were used to compare plaque size (Fig. 2A). Immunohistochemical staining was used to visualize plaques since this approach detects smaller plaques than traditional crystal violet staining, and data are presented on a logarithmic scale to capture the large plaque size differences among viruses. Quantitatively, all sewage reoviruses produced significantly smaller plaques than lab-adapted viruses (Fig. 2B). T2E3 produced smaller plaques compared to other natural isolates, while T3D^PL^ produced the largest plaques. Moreover, T1E1, T2E2, and T2E3 required 10- to 100-fold more virion particles, as measured by the optical density at 260 nm of purified viruses (53), than prototypic strains to achieve a similar number of plaques (Fig. 2B). Overall, all naturally derived reoviruses produced smaller plaques in L929 cells relative to lab-adapted strains.

Cell entry constitutes a common barrier against productive infection by viruses such as SARS-CoV-2, influenza virus, astrovirus, rotavirus, and reovirus (4, 12, 16, 20, 44, 54). The efficiency of cell attachment was therefore assessed for naturally acquired reoviruses. L929 cells were exposed to lab-adapted or sewage reoviruses for 1 hour at 4°C to permit cell attachment without endocytosis (Fig. 2C, 0 hours post-binding) (55). Following extensive washes to remove unbound viruses, cell lysates were subjected to immunoblot analysis using anti-reovirus antibodies and anti-β-actin as loading controls. Quantification of reovirus protein band intensities for cell-associated particles (0 hour post-binding) relative to original inocula (Input) indicated that there were no statistically significant differences in binding efficiencies among the viruses (Fig. 2C, bottom left).

The general dogma based on prototypic lab-adapted strains is that reoviruses can undergo intracellular lysosomal protease-mediated outer capsid uncoating (35, 56 – 59). To determine if naturally acquired reoviruses can also undergo intracellular uncoating, L929 cells synchronously infected at 4°C were then incubated at 37°C to permit endocytosis and exposure of virions to lysosomal proteases. Lysates were collected at multiple timepoints and processed for immunoblot analysis (Fig. 2C and S2A). The kinetics of σ3 degradation and μ1C cleavage to δ were quantified based on the relative band intensities at each timepoint. For lab-adapted reoviruses, σ3 protein degradation progressed very rapidly, achieving ~80% degradation for T3D^PL^ by 2 hours post-infection (hpi) and ~80% for T1L by 4 hpi (Fig. 2C, bottom middle). *De novo* protein synthesis can also be monitored by measuring the increase in full-length μ1, which is cleaved to μ1C during virus outer capsid protein assembly, relative to 0 hpi (60). After 4 hpi, *de novo* protein synthesis began for lab-adapted strains. Compared to prototypic strains, σ3 degradation was delayed for the naturally acquired isolates, ranging between 20% and 40% at 2 hpi and only achieving 60%–70% σ3 degradation at 6 hpi. In terms of μ1C cleavage, T1L and T3D^PL^ reached 50% μ1C-to-δ conversion between 2 and 4 hpi. In contrast, all four natural reovirus isolates achieved only ~10% μ1C-to-δ cleavage at 4 hpi and slightly over 20% at 6 hpi (Fig. 2C, bottom right). The delayed uncoating of naturally acquired reoviruses correlated with delayed *de novo* protein production of full-length μ1.

Finally, to monitor whether uncoating of naturally derived reoviruses progresses further if given sufficient time, we needed to prevent the confounding variable of *de novo* protein synthesis since lab-adapted viruses replenish μ1C and σ3 so rapidly. Accordingly, uncoating experiments were repeated in the presence of cycloheximide to prevent new protein expression. The natural reovirus isolates did eventually achieve σ3 degradation and μ1C cleavage at 10–18 hpi, albeit at very slow kinetics relative to T3D^PL^ (Fig. S2B). Altogether, the results indicate that naturally circulating reoviruses are very inefficient at intracellular uncoating in L929 cells relative to lab-adapted strains.

### Naturally circulating reoviruses are highly dependent on intestinal proteases for uncoating

**FIG 3:**
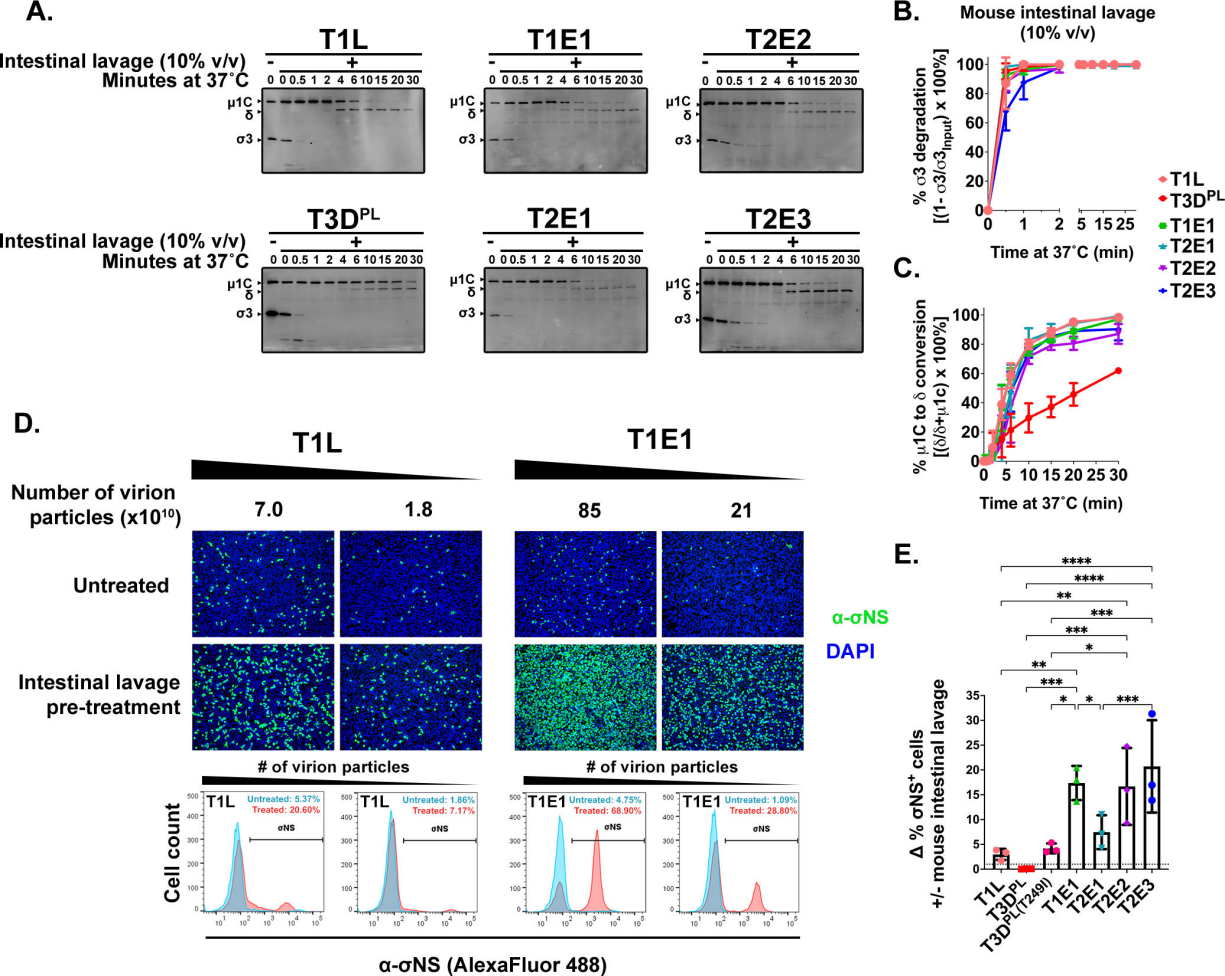
Naturally circulating reoviruses are highly dependent on extracellular intestinal proteases for uncoating. (**A**) An equal number of T1L, T3D^PL^, T1E1, T2E1, T2E2, or T2E3 particles (~2.2 × 10^9^) were mixed with mouse intestinal lavage (10%, vol/vol) in virus dilution buffer before being incubated at 37°C. At the indicated timepoints, samples were processed for immunoblotting using primary anti-reovirus antibodies. (**B**) The kinetics of σ3 degradation were measured by comparing the σ3 protein band intensities over time compared to the no treatment control. (**C**) The kinetics of μ1C-to-δ conversion were determined by measuring the proportion of δ relative to δ + μ1C. Error bars represent the standard deviation from *N* = 3 independent experiments. (**D and E**) T1L, T3D^PL^, T3D^PL(T249I)^, T1E1, T2E1, T2E2, or T2E3 virions (~5%–10% infection by flow cytometry) were mixed with mouse intestinal lavage (10%, vol/vol) in VDB and incubated at 37°C for 30 min. Reactions were neutralized by the addition of an equal volume of FBS-containing media. L929 cells were synchronously infected with either untreated or intestinal lavage-treated virus particles. At 18 hpi, cells were fixed and processed for immunofluorescence microscopy and flow cytometry using anti-σNS antibodies. (**D**) Microscopy (top) and flow cytometry (bottom) data for representative T1L and T1E1. (**E**) Quantification of flow cytometry measurements demonstrating the fold change in percent productively infected cells by intestinal lavage-treated reovirus particles compared to untreated virions. Dots and error bars represent the mean and standard deviation from *N* = 3 biological replicates with two to three virus dilutions each. *P < 0.05, **P < 0.01, ***P < 0.001, ****P < 0.0001 by one-way ANOVA with Tukey’s multiple comparison test.

Given that naturally acquired reoviruses undergo intracellular uncoating more inefficiently than lab-adapted strains, we next distinguished between two possible explanations for this difference: (i) natural reovirus isolates might have evolved to uncoat slowly for some biological benefit such as greater overall stability, or (ii) the necessary conditions for uncoating naturally acquired reoviruses are absent from L929 cell cultures. During intestinal infection, trypsin and chymotrypsin in the intestinal lumen can initiate reovirus uncoating extracellularly (5, 17). Considering the absence of intestinal proteases in L929 cell culture, we wondered if the addition of intestinal proteases could improve the ability of naturally acquired reoviruses to infect L929 cells. Reovirus outer capsid uncoating by gut proteases was assessed *in vitro* by treating equal particle numbers of prototypic and sewage reoviruses with mouse intestinal lavage at 37°C for various periods of time. Quantification of the relative band intensities of σ3, μ1C, and δ at the different timepoints by Western blot analysis revealed that σ3 degradation was extremely fast for all viruses, reaching completion by 2 min (Fig. 3A and B). Interestingly, all viruses except T3D^PL^ had similar μ1C cleavage kinetics, reaching ~80% μ1C-to-δ cleavage by 10 min (Fig. 3A and C). Conversely, T3D^PL^ exhibited delayed μ1C cleavage, achieving ~60% of δ production by 30 min. These findings indicate that naturally acquired reoviruses do not have a general resistance to outer capsid proteolysis but rather are highly sensitive to intestinal proteases and less sensitive to intracellular enzymes compared to L929-adapted T3D^PL^.

Having established that naturally derived reoviruses can uncoat efficiently in the presence of intestinal proteases, it was now possible to test whether inefficient uncoating was the mechanism of poor fitness in L929 cells. If impaired uncoating prevented efficient infection of L929 cells by sewage reoviruses, then prior exposure of wild reoviruses to intestinal proteases should enhance their infectivity toward L929 cells. To test this hypothesis, purified viral particles were mixed with mouse intestinal lavage, incubated at 37°C for 30 min, and then mixed with FBS-containing media to stop further proteolysis. Under these conditions, ISVPs were formed that have σ3 degraded and μ1C cleaved into the membrane-penetrating δ fragment (61), which facilitates virus entry directly through cell surface membranes and permits the onset of infection independently of lysosomal proteolysis (34, 61). L929 cells were exposed to either untreated- or intestinal lavage-treated viral particles for 15–18 hpi to permit establishment of infection, and the percent of productively infected cells was monitored qualitatively by immunofluorescence microscopy (Fig. 3D, top) and quantitatively by flow cytometric analysis (Fig. 3D, bottom) using antibodies that recognize the non-structural σNS protein (62). The number of particles added to L929 cells was normalized among viruses to obtain ~10% productive infection by untreated virions as measured by flow cytometry. L929 cells were also treated with a 1/4-fold dilution of each virus to produce ~2.5% infection by untreated virions and ensure results would not be saturated after lavage pre-treatment. In the representative data comparison of T1L versus T1E1 viruses (Fig. 3D), 12-fold more T1E1 particles were added than T1L to achieve similar infection without lavage pre-treatment consistent with the ~10-fold lower specific infectivity (Fig. 1). Intestinal lavage pre-treatment of T1L virus particles increased the percent of σNS-positive cells by approximately threefold relative to untreated T1L particles. T1E1 pre-treatment with intestinal lavage led to an ~18-fold increase in productive infection, indicating a much higher dependency on extracellular uncoating relative to T1L (Fig. 3D and E). Moreover, since T1E1 became as infectious as T1L when intestinal proteases were available, this indicates that inefficient intracellular uncoating was a major factor for the poor infectivity of T1E1 in L929 cells in the absence of intestinal proteases.

To determine whether intestinal factors increased the infectivity of all naturally derived reoviruses, the experiment in Fig. 3D was performed on all lab-adapted and wild reoviruses. However, to assess T3D^PL^, a confounding factor had to be rectified first: the T3D^PL^ σ1 cell attachment protein is uniquely sensitive to cleavage by chymotrypsin and intestinal lavage treatment (Fig. S3A and B), which renders T3D^PL^ unable to bind cells despite ISVP production. A threonine-to-isoleucine mutation at position 249 of σ1 was previously found to prevent σ1 cleavage (63, 64), and so in order to assess the possible benefits of extracellular ISVP production for T3D^PL^ without the confounding effect of σ1 cleavage, the T249I mutation was introduced into T3D^PL^ σ1 to produce T3D^PL(T249I)^. The σ1 of T3D^PL(T249I)^ was confirmed to not undergo massive cleavage by intestinal lavage (Fig. S3B). Similar to Fig. 3D, flow cytometric analysis was used for all viruses to quantify the increase in productively infected cells by virus particles treated or untreated with intestinal lavage (Fig. 3E). Similar to T1L, infectivity of T3D^PL(T249I)^ increased byapproximately fourfold after pre-treatment with intestinal lavage. Conversely, intestinal lavage treatment increased the infectivity of T1E1, T2E2, and T2E3 by ~18-fold, while T2E1 increased by ~8-fold. T2E1 exhibited higher particle infectivity (Fig. 2B) and *de novo* production of μ1 (Fig. S2A) relative to the other three wild reovirus samples, so it is perhaps less incumbered by poor intracellular uncoating.

Overall, naturally circulating reovirus particles were more dependent on extracellular intestinal proteases than their lab-adapted counterparts, suggesting that the absence of extracellular proteases in cell culture represents a major barrier toward optimal infection of L929 cells by naturally acquired reoviruses.

### Lab-adapted versus naturally derived reoviruses exhibit differential sensitivity to host proteases

Having discovered that naturally acquired reoviruses are more dependent on intestinal proteases to infect L929 cells than lab-adapted strains (Fig. 3), the next question was whether these reoviruses were differentially susceptible to specific intestinal proteases. Most mammalian species can be infected by reoviruses through the digestive tract (24). Within the intestinal lumen, both trypsin and chymotrypsin can convert prototypic reoviruses into ISVPs (5, 17). However, trypsins generally cleave sites with positively charged lysine and arginine residues, while chymotrypsins tend to cleave at the carboxy-terminal of aromatic residues (65). Moreover, trypsin and chymotrypsin from different species exhibit distinct proteolytic preferences depending on the amino acids surrounding the cleavage sites (65, 66), and it remains to be explored whether reoviruses exhibit differential sensitivities to gut proteases from distinct hosts. Since our naturally derived reovirus isolates were obtained from the Edmonton municipal wastewater system, there was a high probability that they originated from human, bovine, or porcine sources, given the sizeable human and livestock populations in the province of Alberta. Accordingly, experiments were designed to establish if lab-adapted and naturally derived reoviruses exhibit unique sensitivity to trypsin versus chymotrypsin gut proteases and also to determine if there were explicit preferences for gut proteases of specific host species origins.

**FIG 4:**
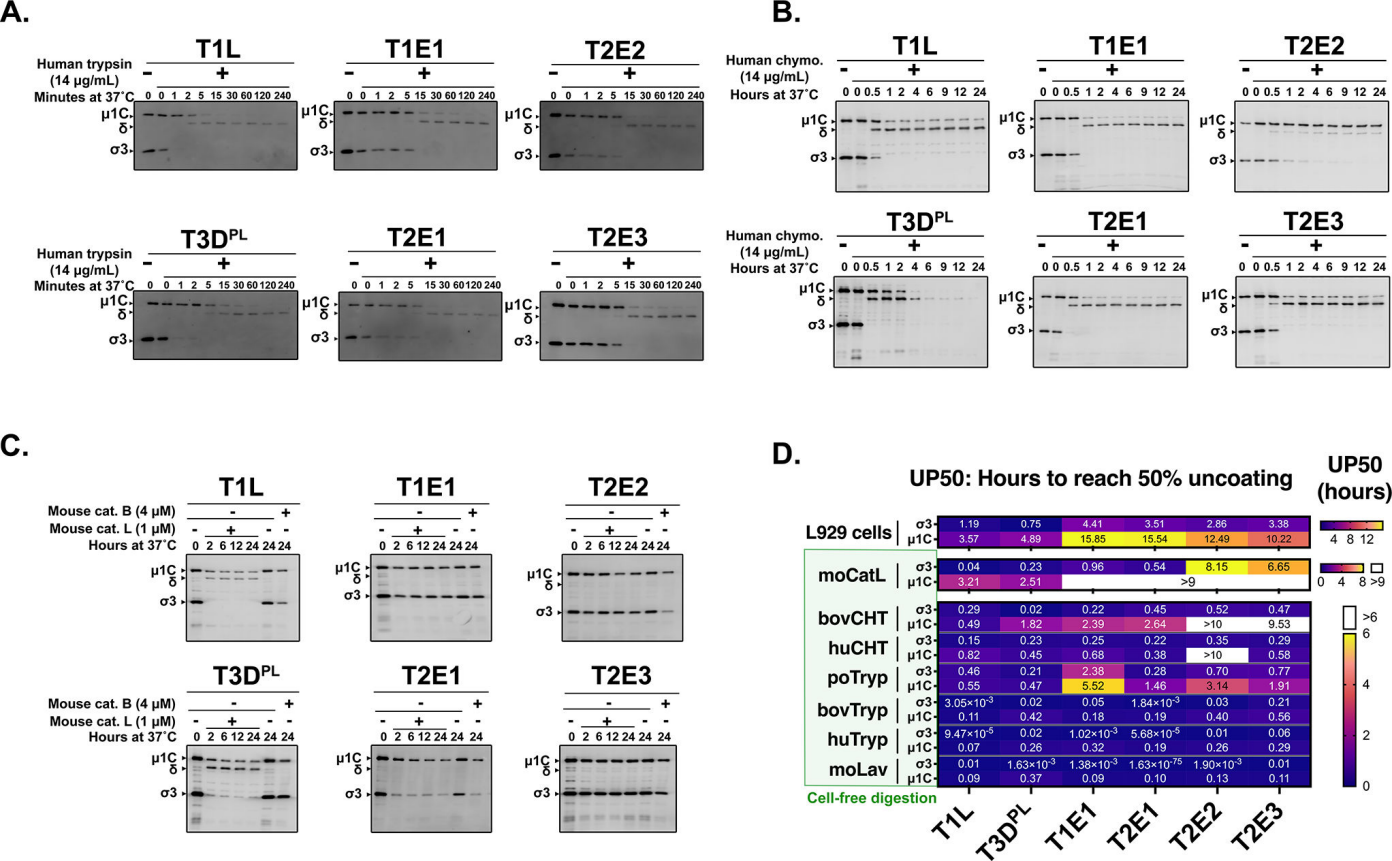
Lab-adapted versus naturally derived reoviruses exhibit differential susceptibility to host proteases. (**A and B**) An equal number of T1L, T3D^PL^, T1E1, T2E1, T2E2, or T2E3 particles (~2.2 × 10^9^) were mixed with recombinant intestinal proteases (14 µg/mL) before being incubated at 37°C. At the indicated timepoints, samples were processed for immunoblotting using anti-reovirus antibodies. (**A**) Blots with human trypsin (14 µg/mL). (**B**) Blots with human chymotrypsin (14 µg/mL). (**C**) Similar to (**A**) and (**B**), except proteolytic reactions were carried out with recombinant mouse cathepsin B (4 µM) or mouse cathepsin L (1 µM). (**D**) The σ3 degradation was measured by comparing σ3 protein band intensities relative to virus alone, while μ1C-to-δ conversion was assessed as the proportion of δ relative to δ + μ1C. Uncoating kinetics curves were generated by calculating percent σ3 degradation or percent μ1C-to-δ cleavage over time. UP50 represents the time it takes to reach 50% digestion as calculated by the Nonlinear Fit-Variable Slope (four parameters) analysis function in Prism 9. UP50 values represent the mean from *N* = 3 independent experiments and are presented as heatmaps.

Lab-adapted and naturally acquired reoviruses were exposed to recombinant trypsin or chymotrypsin at 37°C for various timepoints, and processing to ISVPs was measured by Western blot analysis for σ3 protein degradation and μ1C-to-δ conversion. The gut proteases were derived from human, bovine, or porcine origin, but note that porcine chymotrypsin was commercially unavailable and therefore not included in the studies. When exposed to human trypsin, σ3 protein degradation and cleavage of μ1C to δ progressed rapidly and comparably for all viruses (Fig. 4A). All viruses were also similarly sensitive to bovine trypsin and mouse intestinal lavage (Fig. S4). Conversely, for human chymotrypsin, there was a clear inability of T2E2 to uncoat relative to the remaining lab-adapted and naturally derived reoviruses (Fig. 4B). All four naturally derived isolates also displayed lower sensitivity to porcine trypsin relative to T1L and T3D^PL^ and to bovine chymotrypsin relative to T3D^PL^ (Fig. S4). To facilitate quantitative comparison of proteolysis kinetics among virus-protease combinations over many independent experiments, the processing of σ3 and μ1C was plotted (Fig. S5), and the “hours needed to achieve 50% uncoating” (uncoating percentage 50, UP50) was calculated for σ3 degradation (UP50_σ3_) or μ1C cleavage (UP50_u1C_). From the UP50 values (Fig. 4D), it is evident that all viruses were exquisitely and similarly sensitive to human and bovine trypsins as well as mouse intestinal lavage. Conversely, rates of σ3 degradation and μ1C cleavage varied among viruses exposed to porcine trypsin, as well as bovine and human chymotrypsins. Overall, the results suggest that, with respect to gut proteases, lab-adapted and naturally acquired reoviruses are generally highly sensitive to either trypsin or chymotrypsin, or both. However, some naturally circulating reoviruses exhibited resistance to cleavage by specific gut proteases.

The naturally derived reoviruses uncoated less efficiently in L929 cells and were more dependent on extracellular proteolysis for productive infection compared to lab-adapted strains, which depend mainly on lysosomal cathepsin L for intracellular uncoating with a minor contribution by cathepsin B (35). To determine whether sewage reovirus isolates were less sensitive to lysosomal proteases, *in vitro* digests were performed on lab-adapted and sewage reoviruses with recombinant mouse cathepsin L or B (Fig. 4C). Digestion with cathepsin L resulted in rapid conversion of T1L and T3D^PL^ into ISVPs with complete σ3 degradation and μ1C-to-δ cleavage reaching 40%–50% within 2 hours at 37°C (Fig. 4C). Interestingly, μ1C processing did not progress further with a longer incubation time, suggesting that other cellular factors or a higher enzyme concentration may be necessary for further μ1C cleavage. In contrast, all naturally acquired viruses were resistant to cathepsin L processing, with their σ3 proteins persisting until 24 hours at 37°C and no sign of μ1C-to-δ conversion (Fig. 4C). However, partial σ3 protein degradation was observed; T2E1 achieved ~80% σ3 processing after 24 hours at 37°C, while T1E1 and T2E2 reached 40%–50% σ3 degradation (Fig. S4D). T2E3 was the most resistant to cathepsin L, with only 10% σ3 processing after 24 hours. With regard to cathepsin B, even at 24 hours of treatment, only ~65% σ3 degradation was achieved for T1L, T2E1, and T2E2 and ~25% for T3D^PL^, T1E1, and T2E3. None of the viruses underwent observable μ1C-to-δ cleavage with cathepsin B. Taken together, these findings suggest that despite rapid processing by most digestive enzymes, naturally acquired reoviruses are incapable of undergoing complete viral uncoating with cathepsins L and B, unlike prototypic lab strains.

### Reovirus adaptation to L929 cells correlates with improved intracellular uncoating

Naturally derived reovirus isolates were more reliant on intestinal proteases for productive infection of tumorigenic L929 cells compared to lab-adapted strains that undergo efficient cathepsin-mediated intracellular uncoating. Therefore, efficient intracellular uncoating could be a lab-adapted trait resulting from passaging in L929 cell cultures devoid of digestive enzymes. To better understand molecular features associated with adaptation toward optimal infection in non-enteric vs intestinal niches, naturally acquired T1E1 reovirus was passaged in cell cultures lacking intestinal proteases to produce new “lab-adapted” viruses. If efficient intracellular uncoating is an adaptive trait required for productive infection of non-intestinal models, then T1E1 progeny viruses with greater viral fitness in L929 cells should also exhibit improved intracellular uncoating.

**FIG 5:**
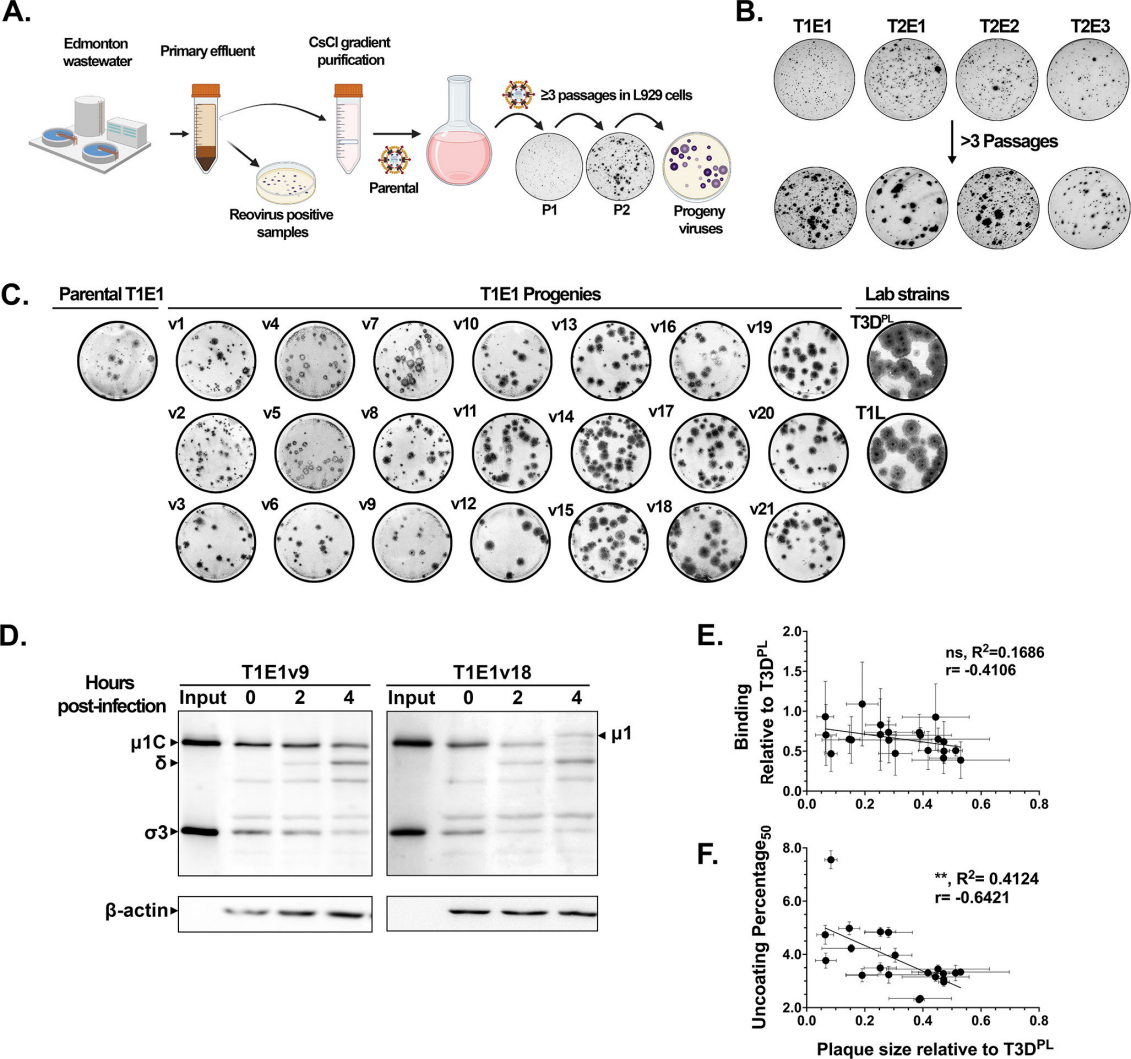
Reovirus adaptation to L929 cells correlates with improved intracellular uncoating. (**A**) Diagram illustrating the process of reovirus isolation from the Edmonton sewage and ensuing adaptation in L929 cells for ≥3 passages. The top 4 images representing plaque size before the three passages are taken from Fig. 2A. (**B**) Plaque size increase of T1E1, T2E1, T2E2, and T2E3 following passage in L929 cells. For the experiment, L929 cells were infected with virus, overlaid with agar-containing media, and incubated at 37°C. Cells were fixed at 4 days post-infection prior to blocking and probing using anti-reovirus primary antibodies. Following incubation with AP-conjugated secondary antibodies, cells were processed for BCIP/NBT staining to produce plaques of infected cells. (**C**) Plaque assays were performed similarly to (**B**), except parental T1E1, T1E1-derived progenies, T1L, or T3D^PL^ was used for infection, and the cells were fixed at 6 days post-infection. Plaque size was quantified using ImageJ. (**D**) L929 cells were synchronously infected with T1E1 progenies for 1 hour at 4°C. Following incubation, cells were washed twice with serum-free media and placed in growth media at 37°C, corresponding to 0 hpi. At the indicated timepoints post-infection, cells were harvested with RIPA buffer and processed for immunoblotting using anti-reovirus and anti-β-actin antibodies. (**E and F**) Binding was calculated from reovirus protein band intensities at 0 hpi (bound) compared to input. The UP50 represents the amount of time (in hours) required to obtain 50% μ1C-to-δ cleavage as calculated by the Nonlinear Fit-Variable Slope (four parameters) analysis function in Prism 9. (**E**) Binding relative to T3D^PL^ and (**F**) UP50 was plotted against plaque size relative to T3D^PL^. Correlation analysis was performed in Prism 9. Error bars represent the standard error for at least *N* = 2 (plaque size) or 3 (binding and UP50) biological replicates. ns >0.1234, **P < 0.0021 by two-tailed correlation analysis.

To identify phenotypic changes associated with increased viral fitness in tumorigenic cell lines, a directed-evolution strategy was employed. T1E1 was serially passaged ≥3 times until P3-5 progeny viruses with variable plaque size emerged (Fig. 5A and B). These progeny viruses (T1E1v1-v21) were then plaque picked, amplified in L929 cells, and purified using Capto Core 700 beads (45). Viral fitness of T1E1 progenies, parental T1E1, and prototypic strains was measured by plaque size analysis in L929 cells, and the progenies were numbered according to sequentially larger plaque sizes. Progeny T1E1 viruses ranged in plaque size from approximately the same size as parental T1E1 (e.g., T1E1v1) to approximately five times larger (T1E1v21) (Fig. 5C and S6A). The majority (≥15 out of 21) of T1E1 progenies produced larger plaques than the parental isolate but remained smaller than T3D^PL^; the largest progeny, T1E1v21, produced plaques ∼50% that of T3D^PL^ (Fig. S6A). These results suggest that the characteristics of naturally derived reoviruses are dynamic and can rapidly change to adapt to new environments within three generations.

To understand which step(s) of virus infection were enhanced in T1E1 progeny, they were assessed for cell binding and uncoating (Fig. 5D). The quantity of cell-associated particles relative to input inoculum revealed variable mean binding (Fig. S6B); however, differences in binding between viruses were not statistically significant, and there was no correlation (*R* ^2^ = 0.1686) between binding efficiency and plaque size of progeny viruses (Fig. 5E). These results indicate that the plaque size increase in L929-adapted T1E1 progenies was not attributable to an improved ability to bind L929 cells. Uncoating kinetics of progeny viruses varied greatly, with T1E1v1 exhibiting the slowest μ1C-to-δ conversion kinetics with an UP50_μ1C_ of ~7.5 hours, which was similar to parental T1E1 (~7 hours) (Fig. S6C). T1E1v2, 4, 7, and 8 displayed slightly faster uncoating kinetics with UP50_μ1C_ at ~5 hours. On the other hand, T1E1v12 and v18 uncoated the fastest among progeny viruses with UP50_μ1C_ of ~2.3 hours, almost as fast as T3D^PL^ (~1.7 hours). Importantly, faster uncoating kinetics (lower UP50_μ1C_ value) in L929 cells had a significant moderate correlation (**, *R* ^2^ = 0.4124, *N* = 3 for uncoating and *N* = 2 for plaque size) with larger plaque size among progeny T1E1 viruses (Fig. 5F). The data suggest that among L929-adapted progenies derived from naturally acquired reoviruses, progenies that produce larger plaques tend to also undergo intracellular uncoating more efficiently. However, intracellular uncoating is unlikely to be the only adaptive trait promoting plaque size in progeny viruses, as demonstrated by the moderate correlation score and the fact that progenies such as T1E1v5 and v6 produced relatively small plaques (~0.15 that of T3D^PL^) despite moderately fast uncoating (UP50_μ1C_ ~3.1 hours). Nevertheless, these results strongly suggest that intracellular uncoating represents a common initial barrier for reovirus adaptation to new non-intestinal niches.

### Reovirus adaptation to intracellular uncoating occurs through cathepsin B/L-dependent and -independent mechanisms

Lysosomal cathepsins B and L mediate viral entry of several viruses, including rotavirus, SARS-CoV-2, and prototypic reovirus (4, 35, 67). However, unlike prototypic strains, naturally derived reoviruses were unable to undergo complete uncoating with cathepsin L (Fig. 4C). As L929-adapted T1E1 progenies displayed improved intracellular uncoating compared to the parental isolate (Fig. S6C), these progeny viruses may have acquired the capacity to undergo more efficient cathepsin-mediated viral uncoating, similar to T1L and T3D^PL^. If progeny viruses adapted to use the same lysosomal proteases as prototypic reoviruses, then inhibiting both cathepsins B and L should decrease the uncoating of progeny viruses to a similar extent as T1L and T3D^PL^. If progenies still uncoat without cathepsin B/L, then alternative protease(s) are likely involved.

**FIG 6:**
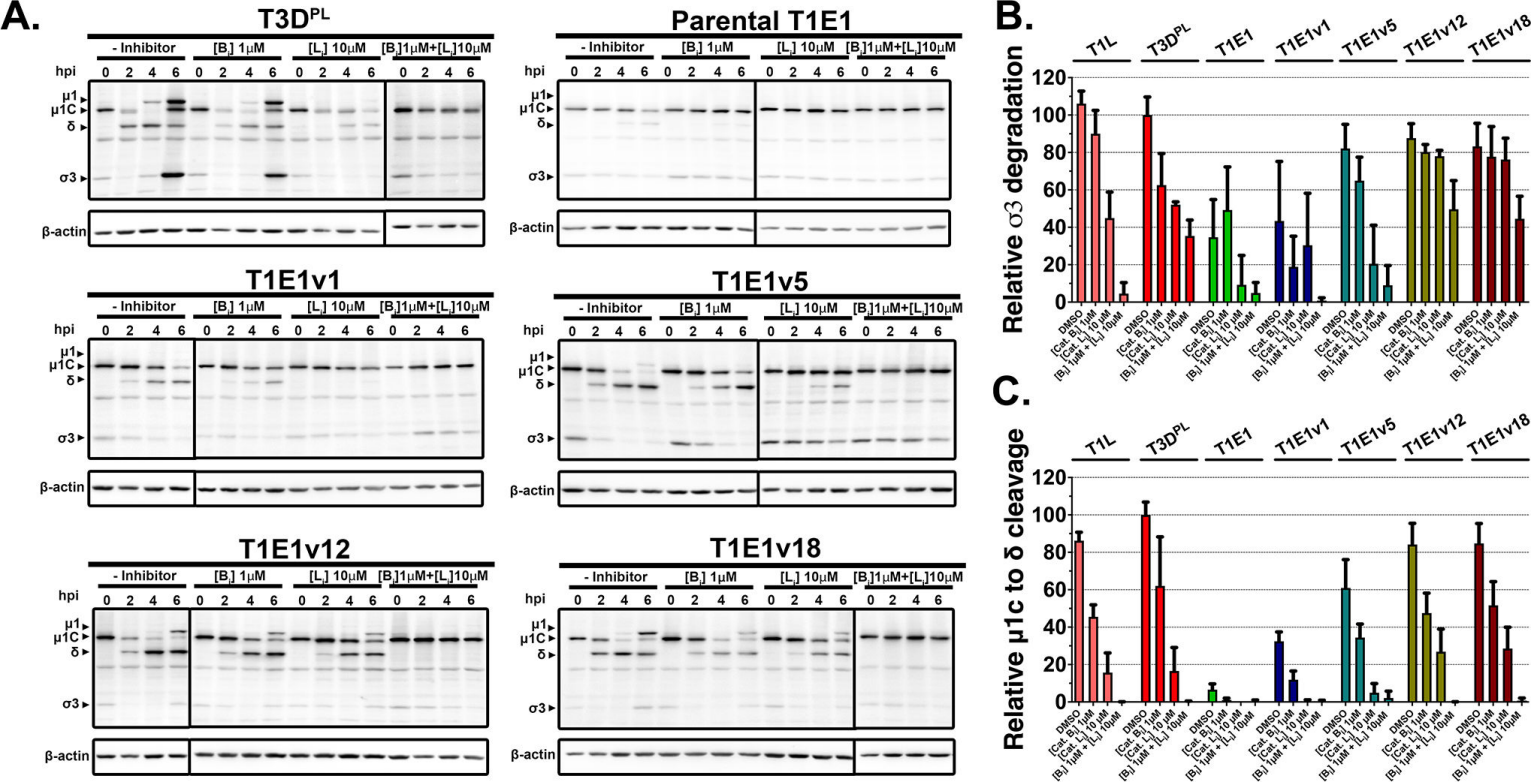
Reovirus adaptation to intracellular uncoating occurs through cathepsin B/L-dependent and -independent mechanisms. (**A**) L929 cells were pre-treated with Cathepsin L Inhibitor III (Cat L_i_, 10 µM), Ca-074 Me (Cathepsin B inhibitor, Cat B_i_, 1 µM), Cat L_i_ + B_i_, or DMSO-untreated control. Cells were then synchronously infected with T1E1, T1E1v1, T1E1v5, T1E1v12, T1E1v18 or prototypic lab strains at 4°C for 1 hour. Following incubation, cells were washed twice with serum-free media and placed at 37°C in growth media containing the corresponding drug treatment. At the indicated timepoints post-infection, cells were harvested with RIPA buffer and processed for immunoblotting using anti-reovirus and anti-β-actin antibodies. (**B and C**) The σ3 degradation was measured by comparing σ3 protein band intensities relative to 0 hpi, while μ1C-to-δ conversion was assessed as the proportion of δ relative to δ + μ1C. The uncoating data were then analyzed by the Nonlinear Fit-Variable Slope (four parameters) analysis function in Prism 9, and the AUC was calculated for each virus as a proxy for total uncoating progression over the course of 6 hours. Values are presented as percentages of DMSO-treated T3D^PL^ AUC in terms of (**B**) σ3 degradation or (**C**) μ1C-to-δ conversion. Error bars correspond to the standard deviation from *N* = 3 biological replicates.

To characterize the intracellular uncoating of progeny viruses, four representative progeny viruses with variable uncoating kinetics and fitness were selected (T1E1v1 = slow, v5 = intermediate, v12 and v18 = fast) to perform uncoating assays in the presence of inhibitors against cathepsin L (10 µM Cathepsin L Inhibitor III, Cat L_i_), cathepsin B (1 µM CA-074 Me, Cat B_i_), or both cathepsins (Cat L_i_ + B_i_) with DMSO treatment serving as negative control. Slow-uncoating parental T1E1 and fast-uncoating prototypic T1L and T3D^PL^ were included for comparison. L929 cells were pre-treated with drugs at concentrations previously reported to exclude cell cytotoxicity for 1 hour at 37°C before being synchronously infected with reoviruses in the continued presence of cathepsin inhibitor(s) or DMSO (35, 68). At various timepoints post-infection, lysates were collected for Western blot analysis (Fig. 6A). Relative band intensities of reovirus outer capsid proteins were used to monitor uncoating progression with the different treatments (Fig. S7). The uncoating data were then analyzed by non-linear regression to generate best-fit curves, and the area under the curve (AUC) was calculated for each virus as a proxy for total uncoating progression over the course of 6 hours. Values were presented as a percentage of the AUC of DMSO-treated T3D^PL^ for both σ3 degradation and μ1C-to-δ conversion.

In the absence of inhibitors, lab strains T1L and T3D^PL^ had similar σ3 degradation and μ1C-to-δ cleavage as fast-uncoating T1E1v12 and v18 progenies with AUC values ranging between 80% and ~105% of untreated T3D^PL^ (Fig. 6B and C). In contrast, σ3 degradation and μ1C cleavage of parental T1E1 were 35% and ~5% compared to T3D^PL^, respectively, while slow-uncoating T1E1v1 and intermediate-uncoating v5 had intermediate uncoating AUC values. Cathepsin L inhibition reduced the uncoating of both T1L and T3D^PL^ more than cathepsin B inhibition alone, but Cat L_i_ + B_i_ treatment maximally abrogated σ3 degradation and μ1C-to-δ cleavage. These findings are consistent with the dominant role of cathepsin L and the supporting role of cathepsin B in the uncoating of prototypic lab-adapted reoviruses (35). However, the T3D^PL^ lab strain also appears to depend on other proteases for σ3 degradation since it retained ~35%–40% σ3 degradation without cathepsin B/L activities, while T1L was completely inhibited (<5%). Cathepsin B/L inhibitors also completely inhibited outer capsid processing of slow-uncoating parental T1E1, slow-uncoating T1E1v1, and intermediate-uncoating T1E1v5, indicating that these viruses were entirely dependent on cathepsin B/L for the limited uncoating they demonstrated without inhibitors. For the fast-uncoating T1E1v12 and v18 progenies, μ1C-to-δ cleavage also displayed dependency on cathepsins B and L, but strikingly, σ3 degradation was only inhibited ≤50% by cathepsin B/L inhibitors. In other words, it appeared that, similar to T3D^PL^, progenies selected from T1E1 that undergo more rapid uncoating also demonstrated an ability to degrade σ3 in a cathepsin B/L-independent manner. Taken together, the data suggest that improved μ1C-to-δ cleavage of lab-adapted T1E1 progenies was reliant on cathepsin B/L similarly to lab strains, but these progenies also acquired proteolytic sensitivity to lysosomal proteases other than cathepsins B/L for σ3 degradation.

### Polymorphisms in σ3 segregate large and small plaque-forming phenotypes of cell culture-adapted reoviruses and are present at ~0.01% frequency among the parental naturally derived quasispecies

**FIG 7:**
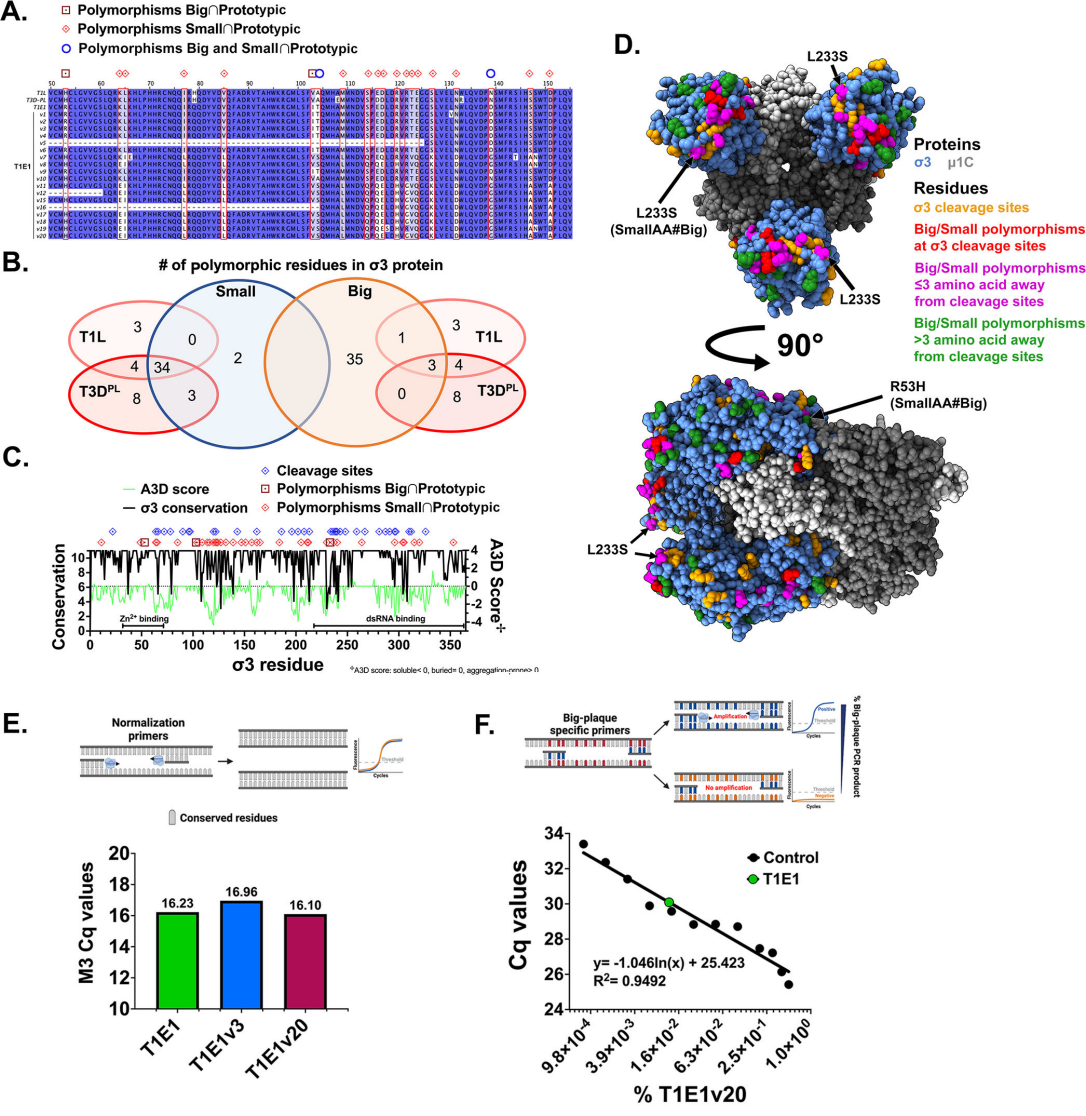
Polymorphisms segregating the large and small plaque-forming phenotypes of cell culture-adapted reoviruses are found in the parental isolate in minor amounts. Whole-genome sequencing was performed on purified reoviruses using the Illumina MiSeq platform. Nucleotide sequences were assembled using the *de novo* SPAdes assembler, and contigs were annotated with NCBI BLASTN (46, 47). Nucleotide sequences were translated into amino acid sequences using the ExPASy translate tool and then aligned with ClustalOmega with default settings. (**A**) Amino acid alignment of the S4 gene-encoded σ3 protein of prototypic T1L-, T3D^PL^-, and T1E1-derived viruses with polymorphic residues segregating progeny viruses into “small” (v1-6, 9, **V10**) and “big” (v7, v8, v11-20) plaque phenotypes highlighted in red rectangles. (**B**) Venn diagram demonstrating the number of polymorphic residues shared between “small” and “big” plaque-forming T1E1 variants and prototypic lab strains. (**C**) Distribution of T1E1 progeny polymorphic residues (red diamond), cleavage sites (blue diamond), conservation (black line), and aggregation propensity (green line) throughout the σ3 linear sequence. Conservation scores (11 = fully conserved) were obtained from analyzing the multiple sequence alignment of 58 full-length amino acid sequences available on GenBank and from our sequencing data with Jalview. Aggrescan3D 2.0 was used to estimate aggregation propensity (A3D Score; soluble residues <0, buried residues = 0, aggregation-prone residues >0) based on the σ3/μ1 heterohexamer crystal structure (60). Putative Zinc^2+^ binding and dsRNA binding domains are also indicated (72, 73). (**D**) Polymorphisms of interest identified in (**A**) were mapped onto the σ3/μ1 heterohexamer crystal structure (60). The σ3 proteins are shown in blue, and the μ1C proteins are shown in gray. Polymorphic residues are colored based on their proximity to known cleavage sites: yellow = conserved cleavage sites (74, 75), red = polymorphic cleavage sites (74, 75), magenta = polymorphisms ≤3 amino acids away from cleavage sites, or green = polymorphisms >3 amino acids away from cleavage sites. (**E**) RNA was extracted from L929 cells infected with either T1E1v3, T1E1v20, or T1E1 and diluted until the levels of viral M3 were equalized as measured by qPCR. (**F**) Normalized T1E1v20 RNA was diluted in T1E1v3 RNA to a final concentration of 0%–1.0% of total viral RNA. A standard curve was generated by qPCR with specific primers recognizing only the T1E1v20 S4. The Cq of parental T1E1 (green dot) was plotted on the standard curve.

RNA viruses generally circulate in nature as mixed populations or “quasispecies” due to their error-prone genome replication (4, 69 – 71). As a result, when new selective pressures are encountered, RNA viruses can rapidly adapt through the selection of pre-existing variants with characteristics beneficial in the new environment (4). To characterize genetic changes associated with improved fitness in T1E1 progenies, amino acid polymorphisms were analyzed by whole-genome sequencing. Among all the T1E1 progenies, S3 and M2 genes were identical, while S1, S2, M1, M3, L1, L2, and L3 displayed amino acid changes with no apparent association to improved plaque size (Table S2). However, in the S4 gene that encodes the outer capsid σ3 protein, 39 out of the 365 residues varied among progeny viruses and segregated T1E1-derived viruses into two groups based on plaque size (Table 1; Fig. 7A). Counterintuitively, the group of “small” plaque-forming progenies shared 34 residues with parental T1E1 and both prototypic T1L and T3D^PL^, while the “big” plaque-forming progenies shared only three residues with prototypic strains (Table 1; Fig. 7B). This result does not directly implicate specific polymorphisms with uncoating proficiency but simply suggests that the parental T1E1 isolate displayed genetic heterogeneity. The polymorphisms in “big” plaque-forming progenies that contribute to enhanced lysosomal proteolysis could be any of the three shared with prototypic T1L and T3D^PL^ but absent from “small” plaque-forming progenies, or they might be a combination of polymorphisms unique to “big” plaque-forming progenies. Regardless, as the only genetic features segregating “small” versus “big” plaque-forming progenies, the results indicate that changes in S4/σ3 and not the remaining genes/proteins become initially selected among T1E1 progenies with improved intracellular uncoating and plaque size in L929 cells.

**TABLE 1:** Amino acid polymorphisms found in σ3 proteins of T1E1 progeny variants^*a*^

Plaque size rel. to T3D^PL^			0.07	0.08	0.09	0.12	0.15	0.21	0.22	0.22	0.22	0.26	0.33	0.37	0.44	0.44	0.46	0.47	0.49	0.53		
Protein/gene	Position	T1E1	v1	v2	v3	v4	v5	v6	v7	v8	v9	v10	v11	v12	v15	v16	v17	v18	v19	v20	T1L	T3D^PL^
σ3/S4	49	*V*	–^*b*^	*V*	*V*	*V*	–	*V*	**I**	**I**	*V*	*V*	**I**	–	**I**	–	**I**	**I**	**I**	**I**	*V*	*V*
	53	*R*	*R*	*R*	*R*	*R*	–	*R*	**H**	**H**	*R*	**H**	**H**	–	**H**	–	**H**	**H**	**H**	**H**	**H**	**H**
	64	*K*	*K*	*K*	*K*	*K*	–	*K*	*K*	**E**	*K*	*K*	**E**	**E**	**E**	–	**E**	**E**	**E**	**E**	*K*	*K*
	65	*L*	*L*	*L*	*L*	*L*	–	*L*	**I**	**I**	*L*	*L*	**I**	**I**	**I**	–	**I**	**I**	**I**	**I**	*L*	*L*
	77	*I*	*I*	*I*	*I*	*I*	–	*I*	**L**	**L**	*I*	*I*	**L**	**L**	**L**	–	**L**	**L**	**L**	**L**	*I*	*I*
	85	*V*	*V*	*V*	*V*	*V*	–	*V*	**L**	**L**	*V*	*V*	**L**	**L**	**L**	–	**L**	**L**	**L**	**L**	*V*	*V*
	103	*I*	*I*	*I*	*I*	*I*	–	*I*	**V**	**V**	*I*	**V**	**V**	**V**	**V**	–	**V**	**V**	**V**	**V**	**V**	**V**
	104	*T*	*T*	*T*	*T*	*T*	–	*T*	**S**	**S**	*T*	A	**S**	**S**	**S**	–	**S**	**S**	**S**	**S**	A	A
	109	*M*	*M*	*M*	*M*	*M*	–	*M*	**L**	**L**	*M*	*M*	**L**	**L**	**L**	**L**	**L**	**L**	**L**	**L**	*M*	*M*
	114	*S*	*S*	*S*	*S*	*S*	–	*S*	**Q**	**Q**	*S*	*S*	**Q**	**Q**	**Q**	**Q**	**Q**	**Q**	**Q**	**Q**	*S*	*S*
	116	*E*	*E*	*E*	*E*	*E*	–	*E*	*E*	**Q**	*E*	*E*	**Q**	**Q**	**Q**	**Q**	**Q**	**Q**	**Q**	**Q**	*E*	D
	117	*D*	*D*	*D*	*D*	*D*	–	*D*	Q	**E**	*D*	*D*	**E**	**E**	**E**	**E**	**E**	**E**	**E**	**E**	*D*	*D*
	118	L	L	L	L	L	–	L	L	L	L	L	L	L	L	L	L	L	S	L	L	L
	120	*R*	*R*	*R*	*R*	*R*	–	*R*	*R*	**H**	*R*	*R*	**H**	**H**	**H**	**H**	**H**	**H**	**H**	**H**	*R*	*R*
	122	*R*	*R*	*R*	*R*	*R*	–	*R*	*R*	*R*	*R*	*R*	**G**	**G**	**G**	**G**	**G**	**G**	*R*	**G**	*R*	*R*
	123	*T*	*T*	*T*	*T*	*T*	–	*T*	**V**	**V**	*T*	*T*	**V**	**V**	**V**	**V**	**V**	**V**	**V**	**V**	*T*	*T*
	124	*E*	*E*	*E*	*E*	*E*	–	*E*	**Q**	**Q**	*E*	*E*	**Q**	**Q**	**Q**	**Q**	**Q**	**Q**	**Q**	**Q**	*E*	*E*
	127	*S*	*S*	*S*	*S*	*S*	*S*	*S*	**K**	**K**	*S*	*S*	**K**	**K**	**K**	**K**	**K**	**K**	**K**	**K**	*S*	*S*
	131	L	V	L	L	L	L	L	L	L	L	L	L	L	L	L	L	L	L	L	L	L
	132	*N*	*N*	*N*	*N*	*N*	*N*	*N*	**D**	**D**	*N*	*N*	**D**	**D**	**D**	**D**	**D**	**D**	**D**	**D**	*N*	*N*
	139	*D*	*D*	*D*	*D*	*D*	*D*	*D*	**G**	**G**	*D*	N	**G**	**G**	**G**	**G**	**G**	**G**	**G**	**G**	N	N
	144	S	S	S	S	S	S	S	T	S	S	S	S	S	S	S	S	S	S	S	S	S
	147	*S*	*S*	*S*	*S*	*S*	*S*	*S*	**A**	**A**	*S*	*S*	**A**	**A**	**A**	**A**	**A**	**A**	**A**	**A**	*S*	*S*
	148	S	S	S	S	S	S	S	N	N	S	S	S	S	S	S	S	S	N	S	S	S
	151	*D*	*D*	*D*	*D*	*D*	*D*	*D*	*D*	*D*	*D*	*D*	**A**	**A**	**A**	**A**	**A**	**A**	*D*	**A**	*D*	*D*
	157	*D*	*D*	*D*	*D*	*D*	*D*	*D*	**E**	**E**	*D*	*D*	**E**	**E**	**E**	**E**	**E**	**E**	**E**	**E**	*D*	*D*
	162	*K*	*K*	*K*	*K*	*K*	*K*	*K*	**Q**	**Q**	*K*	*K*	**Q**	**Q**	**Q**	**Q**	**Q**	**Q**	**Q**	**Q**	*K*	*K*
	165	*Q*	*Q*	*Q*	*Q*	*Q*	*Q*	*Q*	**R**	**R**	*Q*	*Q*	**R**	**R**	**R**	**R**	**R**	**R**	**R**	**R**	*Q*	*Q*
	184	*M*	*M*	*M*	*M*	*M*	*M*	*M*	**L**	**L**	*M*	*M*	**L**	**L**	**L**	**L**	**L**	**L**	**L**	**L**	*M*	*M*
	199	G	G	G	G	G	S	G	G	G	G	G	G	G	G	G	G	G	G	G	G	G
	202	R	R	R	R	R	R	R	K	K	R	R	R	R	R	R	R	R	K	R	R	R
	205	*Q*	*Q*	*Q*	*Q*	*Q*	*Q*	*Q*	**H**	**H**	*Q*	*Q*	**H**	**H**	**H**	**H**	**H**	**H**	**H**	**H**	*Q*	*Q*
	211	*D*	*D*	*D*	*D*	*D*	*D*	*D*	**E**	**E**	*D*	*D*	**E**	**E**	**E**	**E**	**E**	**E**	**E**	**E**	*D*	*D*
	212	*S*	*S*	*S*	*S*	*S*	*S*	*S*	***P***	***P***	*S*	*S*	***P***	***P***	***P***	***P***	***P***	***P***	***P***	***P***	*S*	*S*
	215	S	S	S	S	S	S	S	S	N	S	S	S	S	S	S	S	S	N	S	S	S
	227	E	E	E	E	E	E	E	E	E	E	G	E	E	E	E	E	E	E	E	E	E
	230	*H*	*H*	*H*	*H*	*H*	*H*	*H*	**S**	**S**	*H*	*H*	**S**	**S**	**S**	**S**	**S**	**S**	**S**	**S**	*H*	*H*
	233	*L*	*L*	*L*	*L*	–	*L*	*L*	*L*	**S**	*L*	*L*	**S**	**S**	**S**	**S**	**S**	**S**	**S**	**S**	**S**	**S**
	237	*A*	*A*	*A*	*A*	–	*A*	*A*	**V**	**V**	*A*	*A*	**V**	**V**	**V**	**V**	**V**	**V**	**V**	**V**	*A*	*A*
	238	Y	Y	Y	Y	–	Y	H	Y	Y	Y	Y	Y	Y	Y	Y	Y	Y	Y	Y	Y	Y
	240	R	R	R	R	–	R	R	R	R	R	K	R	R	R	R	R	R	R	R	K	K
	264	I	I	I	I	–	I	I	L	L	I	I	I	I	I	I	I	I	I	I	I	I
	282	M	M	M	M	–	M	M	M	V	–	M	M	M	M	M	M	M	M	M	M	M
	296	*R*	*R*	*R*	*R*	–	*R*	*R*	**K**	**K**	–	*R*	**K**	**K**	**K**	**K**	**K**	**K**	**K**	**K**	*R*	*R*
	297	*K*	*K*	*K*	*K*	–	*K*	*K*	**R**	**R**	–	*K*	**R**	**R**	**R**	**R**	**R**	**R**	**R**	**R**	*K*	*K*
	301	A	A	A	A	–	A	A	A	T	–	A	A	A	A	A	A	A	T	A	S	A
	304	*H*	*H*	*H*	*H*	–	*H*	*H*	**N**	**N**	–	*H*	**N**	**N**	**N**	**N**	**N**	**N**	**N**	**N**	*H*	*H*
	305	*A*	*A*	*A*	*A*	–	*A*	*A*	**I**	**I**	–	*A*	**I**	**I**	**I**	**I**	**I**	**I**	**I**	**I**	*A*	*A*
	315	*L*	*L*	*L*	*L*	–	*L*	–	**I**	**I**	–	*L*	**I**	**I**	**I**	**I**	**I**	**I**	**I**	**I**	*L*	*L*
	320	*M*	*M*	*M*	*M*	–	*M*	–	**I**	**I**	–	*M*	**I**	**I**	**I**	**I**	**I**	**I**	**I**	**I**	*M*	*M*
	325	N	N	N	N	–	N	–	T	N	–	N	N	N	N	N	N	N	N	N	N	N
	353	*N*	*N*	*N*	*N*	–	*N*	–	**G**	**G**	–	*N*	**G**	**G**	**G**	**G**	**G**	–	**G**	**G**	D	*N*

^*a*^ Bold: polymorphisms associated with the big plaque phenotype; italic: polymorphisms associated with the small plaque phenotype.

^*b*^ "–”, sequence unavailable.

Many pressures are likely involved in determining the plasticity of the σ3 protein. The reovirus σ3 protein forms the outermost capsid layer that gets removed first during viral uncoating, so the sequence and structure of σ3 are confined by the need to properly assemble while being sensitive to protease-mediated disassembly (17, 35, 74, 76). In addition to being a major structural component, the reovirus σ3 protein is an immunomodulatory molecule through its dsRNA binding capacity (41, 72) and is therefore likely confined by additional sequence and structure parameters that maintain this secondary function. Other factors *in vivo*, such as antibody binding, might further impact σ3 protein diversification. To better understand the plasticity of σ3 divergence, the σ3 polymorphic residues were mapped onto amino acid conservation data and the 3D structure of the protein (60). The full-length σ3 amino acid sequence of 58 reovirus isolates available on the NCBI GenBank and our naturally derived reoviruses were used to calculate conservation scores for each residue with Jalview (77, 78). Furthermore, the aggregation propensity of each σ3 residue was calculated using Aggrescan3D 2.0 (A3D) and the σ3-μ1C complex 3D structure as an estimation of surface exposure (Fig. 7C, green line; A3D score <0 represents soluble residues, 0 = buried residues, >0 indicates aggregation-prone residues) (60, 79). Residues of T1E1 progenies that segregated with “big” versus “small” plaque size and were distinct from (Fig. 7C, red diamonds) or shared with (Fig. 7C, red squares) T1L and T3D^PL^ were concentrated in the highly divergent and soluble region between positions 100 and 170, with some located throughout the entire sequence, including in the putative zinc binding (residues 51–71) and dsRNA binding (residues 218–365) domains of σ3 (74, 78). These findings support that σ3 genomic plasticity is restrained to specific regions and enriched among surface-exposed regions. Indeed, when mapped onto the σ3-μ1C heterohexamer crystal structure, 31 out of the 39 T1E1 progeny polymorphisms were surface exposed, and 5 of these were at known proteolytic cleavage sites (Table 2; Fig. 7D) (74 – 76). Furthermore, 19 polymorphic residues were within 3 amino acids of cleavage sites and could affect proteolytic digestion since proteases are selective of residues directly before and after their recognition sites (80). For example, cathepsin B has preferences for glycine in P3′ and phenylalanine at the P1′ positions, while cathepsin L has a preference for aromatic residues at P2 (80). Consequently, most of the amino acid polymorphisms in the σ3 protein have the potential to affect protease recognition and, therefore, intracellular uncoating and viral fitness.

**TABLE 2:** Location and characteristics of T1E1 progeny polymorphic residues

Position	Small	Big	Cleavage^*a*^	Near cleavage	A3D score^*b*^
11	V	I			0.00
49	V	I			0.00
53	R	H			−1.57
64	K	E	X	X	−2.98
65	L	I		X	−2.08
77	I	L		X	0.00
85	V	L			0.00
103	I	V			0.00
104	T	S			−0.26
109	M	L			−1.19
114	S	Q			−2.12
116	E	Q			−3.64
117	D	E		X	−3.61
120	R	H	X	X	−4.36
122	R	G	X	X	−3.26
123	T	G		X	−2.50
124	E	Q		X	−2.98
127	S	K			−0.73
132	N	D			−1.43
139	D	G			−1.50
147	S	A			−0.58
151	D	A			−0.80
157	D	E			−2.49
162	K	Q	X		−2.22
165	Q	R		X	−1.76
184	M	L		X	0.00
205	Q	H		X	−1.83
211	D	E		X	−1.45
212	S	P		X	−1.37
230	H	S			−2.95
233	L	S		X	−1.01
237	A	V		X	−1.27
264	L	L		X	0.00
296	R	K	X	X	−3.41
297	K	R		X	−3.12
304	H	N			−1.68
305	A	I			−1.25
315	L	I		X	0.00
320	M	I			0.00
353	N	G			−1.22

^*a*^ Cleavage: reovirus σ3 proteolytic cleavage site (67, 76).

^*b*^ A3D score: Aggrescan3D score—aggregation propensity. Soluble residues <0, buried residues = 0, aggregation-prone residues >0 (71).

The pattern of parallel evolution demonstrated by big plaque-forming progenies suggested that they were either derived from the same rare subpopulation that was already present in the original quasispecies or produced during early culture adaptation. To examine if big plaque-forming variants were present in the parental T1E1 isolate, whole-genome sequence data of T1E1 were re-analyzed for the presence of individual reads containing the big plaque-forming sequence. Most polymorphic regions were sequenced ≥40 times, but all corresponded to parental/small plaque-forming sequences. Thus, the low depth of the sequencing data only suggested that big plaque-associated polymorphisms did not comprise the parental T1E1 quasispecies at a frequency higher than 1:40. To achieve deeper depth, an RT-qPCR approach was applied to detect big plaque-forming variants with greater sensitivity. High-resolution melt (HRM) primers were designed to anneal to conserved S4 sequences flanking a polymorphism-rich region. The resulting amplification products would therefore differ in GC content and produce distinct melt peaks depending on the frequency of big and small plaque S4 genomic sequences. For the experiment, RNA was extracted from L929 cells infected with either parental T1E1, the representative small plaque variant T1E1v3, or the representative big plaque variant T1E1v20. The amount of viral RNA for T1E1, T1E1v3, and T1E1v20 was equalized by RT-qPCR using primers specific for a conserved viral gene segment, M3 (Fig. 7E). Then, to generate control samples with established ratios of big versus small plaque sequences, equalized RNA samples of T1E1v3 and T1E1v20 were mixed in various proportions between 0% and 100%. The melt peak for the 100% purified big plaque sequence was 78.0°C, while the 100% small plaque variant produced a melt peak at 83.0°C (Fig. S8). A small 78.0°C peak could be observed with a 10% big plaque variant, but the parental T1E1 isolate was indistinguishable from a 0% big plaque variant consisting of only small plaque sequence. Thus, HRM analysis could only indicate that big plaque-forming viruses represented less than 10% of the parental T1E1 population, but it could not reveal whether big plaque-forming polymorphisms were present at <10% or absent altogether in the parental T1E1 quasispecies.

To distinguish the presence or absence of big plaque variants in the parental effluent sample with even more sensitivity, qPCR primers recognizing big plaque but not small plaque/parental S4 sequences were designed. Since primer annealing and polymerase activity are heavily dictated by hybridization of the 3′ nucleotides, the 3′ end of primers specific for big plaque variants was designed to anneal polymorphic regions of big plaque S4 sequences. To generate a standard curve of cycle threshold (Cq) versus percent of big plaque polymorphisms (Fig. 7F), equalized viral RNA samples from big plaque variant T1E1v20 and small plaque variant T1E1v3 were mixed such that the big plaque polymorphisms represented from 0.00% to 0.10% of total viral RNA. The Cq of parental T1E1 alone was then plotted on the standard curve and deduced to contain ~0.01% big variant sequence. Although big plaque polymorphisms were detectable in the minimally propagated parental T1E1, they may not necessarily be present at the same ~0.01% frequency in the original wastewater sample. However, given the large number of polymorphisms, it is unlikely to have arisen over two passages and was likely present in the original sewage sample at even lower frequency, and detecting it would require a large amount of the original samples, which are currently unavailable. Nevertheless, the data suggest that big plaque-forming variants existed in the minimally propagated T1E1 quasispecies at ~0.01% frequency and were likely further selected during cell culture passaging due to their ability to uncoat intracellularly and infect L929 cells more efficiently.

Finally, given that the parental T1E1 isolate was composed of both “small” and “big” progeny σ3 sequences, protein BLAST and phylogeny analysis were conducted to determine if the T1E1 progenies were most similar to each other versus other previously sequenced reoviruses. Focusing on the 16 most closely homologous σ3 sequences, there was clear segregation between the small and big plaque progenies. For example, the σ3 sequence of small-plaque progenies was most similar to reoviruses isolated in 2017 from USA bats and a human in France, while the σ3 sequence of big-plaque progenies most closely resembled a reovirus isolated in 2019 from USA bats (Fig. S9). Thus, the single T1E1 reovirus effluent sample already demonstrated the σ3 diversity exhibited between geographic, timeframe, and host variation. Note that it is not possible to distinguish if this diversity was reinstated through *de novo* mutations among the Edmonton reovirus quasispecies or if the T1E1 sample was literally composed of virus particles brought from distant locations and/or hosts.

## DISCUSSION

**FIG 8:**
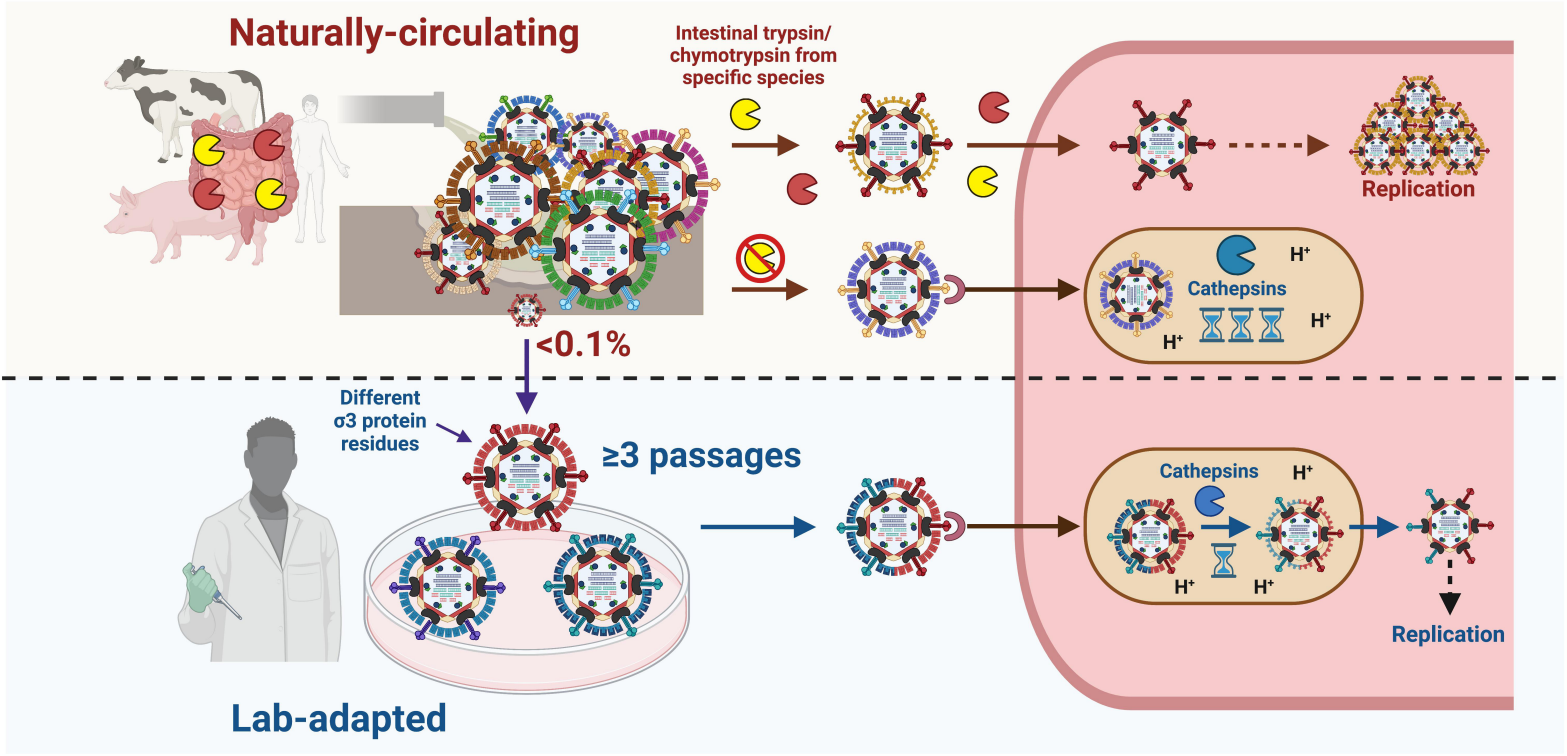
Reovirus adaptation to cell culture involves the selection of genomic variants susceptible to intracellular uncoating. Model summarizing the findings described in this study. Naturally circulating reoviruses rely mainly on trypsin and chymotrypsin present within the intestinal lumen of specific mammalian species to convert virions into ISVP prior to infection. Intracellular uncoating of naturally derived reoviruses is inefficient, and cell-culture passaging results in rapid selection of rare genetic variants with enhanced susceptibility to intracellular proteases. Figure created using Biorender.com.

Mammalian gastrointestinal and respiratory tracts are rich in extracellular host factors that viruses, such as reovirus, rotavirus, astrovirus, and SARS-CoV-2, have evolved to use for promoting infection (4, 16, 20). In this study, using reovirus as a model enteric virus, we examined the genetic and molecular properties of naturally circulating reoviruses versus prototypic lab-adapted strains. Genetic analysis indicated that reoviruses in nature circulate as highly diverse quasispecies (Fig. 1 and 7; Table S1), which may contribute to the high prevalence of reoviruses in virtually all mammalian species (24, 25). Prototypic T1L and T3D^PL^ reoviruses are proficient at infecting murine L929 cells, producing large plaques, and undergoing rapid cathepsin-mediated uncoating (Fig. 2, 4 and 6). In contrast, naturally circulating reoviruses had delayed uncoating, produced smaller plaques on L929 cells, and depended more heavily on intestinal proteases to mediate uncoating and productive infection (Fig. 2 to 4). Thus, natural reovirus infection is likely initiated by ISVP production in the intestinal lumen, while efficient cathepsin-mediated uncoating represents a barrier toward optimal cell culture infection that lab-adapted strains have overcome. Passaging one of the naturally circulating reoviruses in L929 cells rapidly selected for progeny viruses with improved viral fitness, faster intracellular uncoating, and distinct σ3 polymorphisms (Fig. 5 to 7; Tables 1 and 2). The σ3 polymorphisms were present at ~0.01% of the parental quasispecies, demonstrating how the behavior of reovirus as a quasispecies is different from isogenic strains in their capacity to adapt to environmental changes, such as host protease availability. Altogether, this generates a model where reoviruses in nature circulate as quasispecies and rely on specific intestinal enzymes for infection. In response to environmental changes, the tremendous genetic diversity between and within reovirus quasispecies allows it to adapt rapidly by selecting subpopulations susceptible to available proteases (Fig. 8).

### High phenotypic diversity accompanies high genetic diversity

From the many publicly available reovirus sequences representing an assortment of host sources and geographical locations, it is clear that reovirus sequences diverge immensely, but whether these viruses exhibit phenotypic differences has yet to be characterized. Herein, we discover both genetic and phenotypic diversity among independent reovirus samples from the same wastewater site, between these effluent samples and prototypic T1L and T3D^PL^ strains, and even among “small” versus “large” plaque-forming viruses from a single effluent sample. The phenotypic differences, herein focused on sensitivity toward gut and intracellular proteases for uncoating, demonstrate a correlation between genotype and phenotype. While reovirus itself is not pathogenic, one can imagine how the high phenotypic diversity of this experimental model could apply to pathogenic viruses. Our results raise some interesting questions about virus diversity. First, most virus sequences are derived from single hosts with specific bottlenecks; these collections may therefore underrepresent the true diversity of environmental quasispecies. Using effluent samples as a source of virus from multiple hosts represents a powerful strategy to study a wide phenotypic and genetic diversity, at least for environmentally stable viruses like reovirus. However, it seems from our T1E1 analysis that even deep sequencing may underestimate the true diversity since only viruses present at sufficient frequency can be identified by the depth of each read. As single virus purification and sequencing technologies evolve, it will be exciting to learn about the true diversity of reoviruses in wastewater samples.

A second consideration is the challenge of determining whether diversity in a quasispecies arises *de novo* through mutations or represents viruses from distant hosts or geographical locations. For example, within the T1E1 sample, the “small” and “large” plaque-forming virions were more divergent from each other than viruses from other countries. But how can we determine if these viruses evolved in Edmonton or were brought there through transmission and migration of hosts from other geographical areas? Third, aside from mutations, segmented RNA viruses such as reovirus also diversify through reassortment (49, 81). It was evident that the effluent samples did represent reassortment because of the serotype-specific differences among σ1 proteins that do not arise *de novo* but rather come from one of the four serotype lineages. Recent discoveries help explain how viruses can co-infect *in vivo* to produce reassortants despite low virus-cell ratios (49). Specific bacteria strains within the microbiota can promote recombination of poliovirus at low virus-to-cell ratios (81), or extracellular vesicles can deliver clusters of enteric rotavirus and norovirus for more efficient fecal-oral infection than free viruses (82). But where exactly the Edmonton effluent reoviruses were reassorted remains unknown. Given that reoviruses infect many mammals, are there specific hosts that provide more opportunities for reassortment?

### Natural reoviruses are dependent on gut proteases rather than the lysosomal proteases utilized by laboratory strains

The current dogma is that reovirus can be uncoated efficiently either extracellularly by gut proteases or intracellularly in endolysosomes by cathepsins B, L, and S (5, 6, 17, 35, 56). The intracellular uncoating has been demonstrated in a plethora of cultured cells, including polarized epithelial cells (83), as well as *in vivo* (84), but always with lab-adapted T1L or T3D reoviruses. It remained unknown whether naturally circulating reoviruses can also efficiently uncoat using lysosomal proteases or whether they depend heavily on digestive proteases? We asked this question because of the seemingly profound differences between the natural route of enteric viruses enriched in gut proteases compared to cell culture system devoid of gut proteases. The data herein support that, at least for the four naturally derived effluent samples, reovirus seems far less capable of utilizing intracellular proteases than previously thought and relies on digestive enzymes to infect murine L929 fibroblast cells efficiently. In addition to intracellular uncoating, naturally circulating and lab-adapted reoviruses could differ in later stages of infection, such as viral RNA transcription, protein production, assembly, and/or egress, similar to previous findings on prototypic T1L and T3D strains (41, 42, 85). Potential differences in post-uncoating steps would be worth investigating in the future to further our understanding of cultural adaptation. While isogenic viruses have been instrumental in our fundamental understanding of viruses, our findings caution against the use of viruses propagated in culture to derive a comprehensive understanding of what a virus is capable or incapable of doing.

### Diversity supports rapid virus adaptation to new host proteases

Our findings demonstrate how the diversity of reoviruses enables adaptation to new host proteases. Specifically, three rounds of amplification with the T1E1 quasispecies allowed the isolation of strains capable of intracellular uncoating. The relative “ease“ of adapting to a new host protease by reovirus resembles recent findings with SARS-CoV-2, where passaging in cells lacking TMPRSS2 generated mutants with greater sensitivity to cathepsins while retaining the wild-type variant susceptible to TMPRSS2 at a lower proportion (4). It would be interesting to evaluate the adaptability of reovirus toward other host factors required for infection that might vary among the many hosts it infects. For example, vesicular stomatitis virus passaged under variable temperature acquired greater genetic diversity than a lineage grown at constant temperature, which led to a greater capacity to adapt to a higher temperature stress (86). Moreover, a mutant poliovirus with high-fidelity polymerase exhibited lower viral fitness than wild type when faced with additional attenuating mutations due to an inability to mutate and escape the selective pressure (87). Given that reoviruses infect most mammals tested so far, it would be fascinating to know whether genetic diversity contributes to broad host permissivity, or reciprocally, if the broad host range contributes to greater genetic and phenotypic diversity of viruses.

### Sensitivity to type and species source of gut protease varies among naturally circulating reoviruses

Since mammalian gut enzymes differ in specificities and sensitivities to substrates (88), we investigated if reoviruses exhibit differential sensitivity to specific mammalian gut proteases. The *in vitro* digestion results suggested that, with respect to gut proteases, lab-adapted and naturally acquired reoviruses are generally highly sensitive to either trypsin or chymotrypsin, or both. However, some naturally circulating reoviruses exhibited resistance to cleavage by specific gut proteases. This is the first time, to our knowledge, that differences in sensitivity to species source of a given protease have been comprehensively characterized among virus variants. Given that reoviruses infect a variety of mammalian hosts, the wide variation in sensitivities to specific gut proteases might afford reovirus quasispecies a broader compatibility across species. The results raise an interesting question: what rate of proteolysis is most optimal for an enteric virus *in vivo*? Slow proteolysis might unnecessarily delay infection and provide time for host responses, but perhaps rapid proteolysis prior to reaching the gut cells might come with disadvantages. In the future, it would be interesting to create parental viruses with only σ3 from the various effluent strains and determine if differential sensitivity to host-specific proteases correlates with lower or higher propagation and transmission in the corresponding host *in vivo.*

### Adaptation to intracellular uncoating can be achieved through sensitivity to distinct lysosomal proteases

Different reoviruses can utilize distinct lysosomal proteases for uncoating. Cathepsins B and L can efficiently process σ3 and µ1 for lab-adapted T1L and T3D^PL^ reoviruses (35). However, inhibiting cathepsins B and L prevented σ3 degradation for T1L but not T3D^PL^, suggesting that T3D^PL^ can utilize additional lysosomal proteases for σ3 degradation. Similarly, while small plaque-forming T1E1 progenies required cathepsins B and L for the minimal uncoating they could achieve, large plaque-forming T1E1 progenies became independent of these cathepsins for σ3 degradation. These results suggest that viruses can evolve to utilize different lysosomal proteases, which are likely enabled by the variety of host proteases available in lysosomes. Previous studies found that a T3D σ3 mutation at position 354 enabled *in vitro* cathepsin D-mediated σ3 degradation and overcame virus inhibition by cysteine protease inhibitor (E64) and acid-dependent protease inhibition (ammonium chloride) (58, 59). Similarly, large plaque-forming T1E1 progenies had a σ3 polymorphism at position 353 that could endow cathepsin B/L with independent σ3 degradation by creating a cleavage site for cathepsin D or other lysosomal proteases. Overall, previous and current findings suggest that reoviruses can diversely adapt to whichever host proteases are available to achieve disassembly.

Greater sensitivity to lysosomal proteases could be a common adaptive strategy for enteric viruses to meet their proteolytic requirements in non-intestinal environments (67). Host-derived proteases are commonly hijacked by enteric and respiratory viruses to activate viral particles for infection (4, 16, 67). For some clinical rotavirus isolates that do not initially infect continuous cell lines efficiently, culturing in the presence of exogenous trypsin and intracellular cathepsins can gradually enable culture adaptation (12, 67). Human astrovirus is another example of an enteric virus relying on trypsin activation for efficient infection (16, 18), and it would be interesting to know if passaging astroviruses without trypsin could generate mutant viruses with altered protease compatibility. Respiratory viruses like SARS-CoV-2 and influenza virus can undergo proteolysis by trypsin-like proteases (e.g., TMPRSS2 and HAT) expressed on the surface of airway cells but adapt to new proteases under selection (4, 54, 89). Are there any limitations then to whether viruses can adapt toward species or niche-specific proteases?

### Reovirus σ3 protein exhibits regionally specific plasticity despite having high structural and functional constraints

Finally, with a specific focus on the reovirus outer capsid σ3 protein, our analysis of genetic variations among the Edmonton effluent strains, the T1E1 “big” versus “small” plaque progenies, and published sequences revealed that specific regions afford vast variability. The variation is enriched in highly surface-exposed amino acids, which is consistent with pressures imposed on this protein, including the need to commit to the correct structure and µ1 association for assembly and fulfill the secondary roles of σ3 as a dsRNA-binding protein that subdues antiviral signaling. The variability at the σ3 surface might be driven by the need to be compatible with proteases from many hosts, but it is likely also driven by other pressures such as antibody binding. What was interesting was that “small” plaque-forming T1E1 progenies, which uncoated inefficiently, were more genetically similar to prototypic T1L and T3D^PL^ strains that uncoated efficiently. The enhanced uncoating by “big” plaque-forming progenies could be due to: (i) the three polymorphisms shared between the “big” plaque progenies and T1L/T3D^PL^, (ii) the 35 polymorphisms unique to “big” plaque progenies relative to T1L/T3D^PL^ and “small” plaque progenies, or (iii) the lack of the two polymorphisms unique to “small” plaque progenies relative to T1L/T3D^PL^ and “big” plaque progenies. Since σ3 proteolysis must occur prior to µ1C cleavage, the σ3 protein polymorphisms could affect the kinetics of µ1C cleavage indirectly simply by rendering the outer capsid σ3 protein more sensitive to proteolytic degradation (17, 74, 76). Specifically, the σ3 polymorphisms may alter the sequence of proteolytic cleavages and/or pH-dependent conformational changes of the σ3 protein, resulting in earlier exposure of the underlying µ1C for “big” plaque progenies (74, 76). Mutations of the 39 possible polymorphisms were beyond the scope of this study, especially given the high probability that combinations of polymorphisms together impact proteolytic sensitivity and would require endless permutations to reveal. Nevertheless, the findings generally suggest that high sequence similarity does not necessarily implicate phenotypic resemblance. Future characterization of σ3 protein polymorphisms that endow specific protease specificity will enable more tailored analysis of the implication of specific protease utilization on virus behavior in whole animal systems.

## MATERIALS AND METHODS

### Cell lines and viruses

Murine fibroblast L929 cells and rhesus monkey epithelial MA104 cells were purchased from the American Type Culture Collection (ATCC) and maintained in MEM (SIGMA, M4655) supplemented with 5% FBS (Sigma, F1051), 1× nonessential-essential amino acids (Millipore Sigma, M7145), and 1 mM sodium pyruvate (SIGMA, S8636) at 37°C with 5% CO_2_. Testing for mycoplasma contamination was performed using PCR [Applied Biological Materials (abm), G238].

Prototypic reovirus serotypes 1 Lang (T1L) and 3 Dearing (T3D^PL^) were generous gifts from Dr. Terence Dermody (University of Pittsburgh) and Dr. Patrick Lee (Dalhousie University), respectively. Natural isolates of reovirus were obtained from Edmonton primary effluent collected and identified by Xiao-Li Pang (Alberta Provincial Laboratory, Canada). Lab-adapted strains were propagated in adherent L929 cells, while primary effluent samples were passaged once on MA104 cells in the presence of trypsin to produce “seed” virus stocks used for a second passage on L929 cells. All viruses were extracted from infected cell lysates using Vertrel XF (Dymar Chemicals) and purified using the Capto Core 700 in-slurry approach as described previously (45). Purified reovirus particle concentration was estimated by measuring the spectrophotometric optical density at 260 nm with an OD_260_ of 5.42 = 1.13 × 10^13^ particles (90).

### RNA extraction, DNA library prep, and whole-genome sequencing

For RNA extraction, TRIZOL LS (Invitrogen, 10296028) was added to undiluted purified reovirus particles at a 3:1 ratio and mixed vigorously by shaking for 30 s before incubating at room temperature for 5 min. Meanwhile, Phasemaker tubes (Invitrogen, A33248) were prepared by centrifuging at 12,000 × *g* for 30 s at room temperature. After incubation, TRIZOL LS-virus mixtures were transferred into the Phasemaker tubes, and approximately 1/5 of the total volume of chloroform was added. Samples were mixed vigorously by shaking for 30 s and incubated at room temperature for another 5 min before being centrifuged at 12,000 × *g* for 15 min at 4°C. The top aqueous phase was transferred into new RNase-free tubes. Sodium acetate (Thermo, AM9740), glycogen (Thermo, R0551), and cold isopropanol (Fisher, 3510034) were added to final concentrations of 300 mM, 0.1 µg/mL, and 50% (vol/vol), respectively. Samples were mixed gently by flicking before being incubated at −80°C for ≥30 min. After incubation, samples were centrifuged at 12,000 × *g* at 4°C for 15 min. The supernatant was discarded, and the pellet was rinsed twice with cold 75% ethanol by centrifuging at 12,000 × *g* for 15 min at 4°C between washes. An additional dry spin was performed for 3 min at 12,000 × *g* at 4°C, and residual ethanol was removed by pipetting. Samples were air dried for 15–30 min until no residual ethanol remained. The pellet was resuspended in 25 µL of RNase-free water (Fisher, 10977015) and incubated at 65°C for 5 min. RNA was quantified using an ND-1000 NanoDrop spectrophotometer.

To prepare cDNA for library prep, 10 µL (corresponding to 400–800 ng) of extracted RNA per 20 µL reaction was used to perform first-strand cDNA synthesis using Superscript IV reverse transcriptase (Invitrogen, 18090050) and random primers (Fisher, 48190011) following the manufacturer’s protocol. First-strand cDNA products were used as templates for second-strand cDNA synthesis using the *E. coli* DNA polymerase I, RNase H, and DNA ligase enzyme mix (Fisher, A48570), followed by column purification (Invitrogen, K310002) according to the manufacturer’s protocols. DNA libraries for whole-genome sequencing were prepared using the Illumina DNA Prep Kit (Illumina, 20018704) according to the manufacturer’s protocol with the Nextera^TM^ DNA CD Indexes (96 Indexes; Illumina, 20018708). DNA libraries were analyzed for fragment size on an Agilent 2100 Bioanalyzer (Agilent Technologies) using the Agilent High Sensitivity DNA Kit (Agilent, 5067-4626) and concentrations on a Qubit (ThermoFisher) from the University of Alberta Molecular Biology Facility (MBSU) according to the manufacturer’s protocols.

Next-generation sequencing was performed by the University of Alberta High Content Analysis Core on an Illumina MiSeq instrument (Illumina). Sequencing was performed using the MiSeq Reagent Kit v2 flow cell (300 cycles; Illumina, MS-102-2002) with 10 pM loading with 20% PhiX. Sequencing reads were trimmed using Trimmomatic and assembled into contigs using Shovill SPAdes Version 1.1.0 *de novo* assembler tools available on https://usegalaxy.org. The complete workflow used to generate contigs from raw sequencing reads can be found at: https://usegalaxy.org/u/qlin13/w/workflow-constructed-from-history-wilds-sequences/json. Individual contigs were then annotated manually using NCBI-BLASTN. Reovirus sequences for T1E1, T2E1, T2E2, T2E3, and T1E1 progenies have been submitted to GenBank with accession numbers OQ924304-OQ924313 (T1E1), OR074525-OR074534 (T2E1), OR074535-OR074544 (T2E2), OR074545-OR074554 (T2E3), and OR074555-OR074728 (T1E1 progenies), respectively (Table S3).

### Phylogenetic analysis

Nucleotide sequences of mammalian orthoreovirus genes were obtained either through the above-mentioned next-generation sequencing protocol or from the NCBI GenBank database. Multiple sequence alignments were executed using the EMBL-EBI ClustalOmega program (https://www.ebi.ac.uk/Tools/msa/clustalo/) with default settings. Phylogenetic trees of individual viral segments were performed on MEGA 11 using the maximum-likelihood method based on the Tamura-Nei model with 1,000 bootstrap replicates (48).

### Coomassie staining

Purified reovirus particles (~2.5 × 10^10^ particles) were diluted in virus dilution buffer (VDB; 150 mM NaCl, 15 mM MgCl_2_, 10 mM Tris-HCl pH 7.4). Then, 5× protein sample buffer (250 mM Tris-HCl pH 6.8, 5% SDS, 45% glycerol, 9% β-mercaptoethanol, and 0.01% bromophenol blue) was added to each sample to a final concentration of 1×. Samples were boiled at 100°C for 10 min and resolved by sodium dodecyl sulfate-polyacrylamide gel electrophoresis (SDS-PAGE). Gels were then rinsed in ddH_2_O for 5 min at room temperature and stained with Imperial Protein Stain (Thermo, 24615) for 1 hour at room temperature with gentle rocking. Gels were destained in ddH_2_O overnight at room temperature and imaged using the transillumination setting on an ImageQuant LAS4010 Imager (GE Healthcare Life Sciences). Densitometric analysis was performed with ImageQuant TL (GE Healthcare Life Sciences) software, and images were processed for display in Adobe Photoshop.

### Western blot

For standard Western blot analysis, cells were washed once with PBS before harvesting with RIPA lysis buffer [50 mM Tris-HCl pH 7.4, 150 mM NaCl, 1% IGEPAL CA-630 (NP-40), 0.5% sodium deoxycholate] supplemented with protease inhibitor cocktail (P8340, Sigma). Alternatively, purified viruses diluted in VDB were used for serotype determination. To each sample, 5× protein sample buffer (250 mM Tris-HCl pH 6.8, 5% SDS, 45% glycerol, 9% β-mercaptoethanol, and 0.01% bromophenol blue) was added to a final concentration of 1×. Samples were then boiled at 100°C for 10 min before being resolved by SDS-PAGE. Afterward, proteins were transferred onto nitrocellulose membranes using a Trans-Blot Turbo Transfer System (Bio-Rad). Membranes were incubated with block buffer [3% newborn calf serum (NBCS; Gibco, 16010) in TBS-T (Tris-buffered saline-0.1% Tween 20)] for 1 hour at room temperature, followed by probing with the indicated primary antibody diluted in block buffer [rabbit serotype-specific polyclonal antibody (pAb; anti-T1 σ1, anti-T3 σ1, and anti-T2 total virus), 1:500, generous gifts from Dr. Patrick Lee (Dalhousie University); rabbit anti-reovirus pAb, 10,000, also from Dr. Patrick Lee; mouse anti-β-actin mAb, 1:4,000, Cell Signaling Technology 8H10D10] overnight at 4°C. The next day membranes were washed thrice with TBS-T for 5 min each before probing with secondary antibodies in block buffer (HRP-conjugated goat anti-rabbit pAb, 1:10,000, Jackson ImmunoResearch 111-035-144; Alexa Fluor 647-conjugated goat anti-mouse pAb, 1:3,000, Jackson ImmunoResearch 115-605-146) for 1 hour at room temperature. After incubation, membranes were washed three times with TBS-T for 5 min each. Membranes with HRP-conjugated secondary antibodies were incubated with ECL Plus Western Blotting Substrate (Fisher, 32132) according to the manufacturer’s instructions. All membranes were imaged using an ImageQuant LAS4010 Imager (GE Healthcare Life Sciences). Densitometric analysis was performed with ImageQuant TL (GE Healthcare Life Sciences) software, and images were processed for display in Adobe Photoshop. All incubation steps for membranes were done with gentle rocking.

### Virus titering and plaque size analysis

For reovirus plaque analysis, reovirus dilutions in serum-free MEM were added onto confluent monolayers of L929 cells for 1 hour with gentle rocking every 5–10 min. After 1 hour, an agar overlay made of a 2:1:1 ratio of complete MEM media:2× MEM media (Sigma, M0518):2% agar (BD Diagnostics, BD 214010) was added to each well and allowed to solidify at room temperature for 30 min before incubation at 37°C for 4–7 days. After incubation, 4% formaldehyde in PBS was added to the overlay for 1 hour at room temperature. Formaldehyde was then discarded, and agar overlays were gently removed before cells were fixed with methanol for 10 min at room temperature. After discarding the methanol, cells were washed thrice with PBS for 2 min each at room temperature before blocking for 1 hour at room temperature in 3% NBCS/PBS/0.1% Triton X-100. Blocking buffer was then replaced with primary antibodies (rabbit anti-reovirus pAb, 10,000 in block buffer) and incubated overnight at 4°C. The next day, following removal of primary antibodies, cells were washed thrice with PBS for 5–10 min each at room temperature and incubated with secondary antibodies [Alkaline Phosphatase (AP)-conjugated goat anti-rabbit pAb, 1:4,000 in block buffer, Jackson ImmunoResearch 111-055-144] for 1 hour at room temperature. After removal of secondary antibodies, cells were washed thrice with PBS for 5–10 min each at room temperature and washed twice with AP buffer (100 mM Tris-HCl pH 9.5, 100 mM NaCl, 5 mM MgCl_2_) for 2 min each at room temperature. Nitrotetrazolium Blue Chloride (NBT, 300 µg/mL; Sigma, N6639-1G) and 5-Bromo-4-chloro-3-indolylphosphate p-toluidine salt (BCIP, 150 µg/mL; Sigma, B8503-500mg) substrates were diluted in AP buffer and incubated on cells protected from light at room temperature for 10–60 min or until dark purple plaques were visible by eye. Reactions were stopped by the addition of PBS/5 mM EDTA. Plates were then air dried overnight and imaged on an EVOS FL Auto Imaging System (Life Technologies). Plaque area was measured using the “Analyze particles” feature on ImageJ/Fiji (version 1.51).

### Synchronous reovirus infection

Confluent monolayers of L929 cells were pre-chilled at 4°C for 1 hour, followed by addition of reovirus (~1.0 × 10^10^ particles) diluted in serum-free media. Infection was allowed to proceed at 4°C for 1 hour to permit virus attachment. The virus inoculum was then removed, cells were washed twice with serum-free MEM to remove unbound virus, and they were incubated in complete growth media for the indicated timepoints at 37°C. For experiments including drug treatments, cells were pre-treated for 1 hour at 37°C with 10 µM Cathepsin L Inhibitor III (Sigma, 219427), 1 µM CA-074 Me (Sigma, 205531), or dimethyl sulfoxide (DMSO; Sigma, D5879-500ML) prior to pre-chilling the cells at 4°C. Drugs were omitted from the reovirus inoculum but maintained throughout the course of infection at 37°C in the growth media. In the case of cycloheximide (Sigma, C4859-1mL) treatment, 100 µg/mL of drug or DMSO vehicle control was included in the complete growth medium added following viral attachment and washes.

### Intestinal lavage collection

Intestinal lavage samples were prepared by flushing entire murine intestines with 1 mL of PBS, followed by filtering through a 0.45-µM filter to remove debris. To avoid enzymatic inactivation due to freeze-thaw cycles, intestinal lavage samples were aliquoted before flash-freezing in liquid nitrogen. Aliquots were stored at −80°C, and a fresh aliquot was used for each experiment. The total protein concentration of the intestinal lavage samples was determined to be 3.8 mg/mL by the Pierce BCA Protein Assay Kit (Thermo, 232227) according to the manufacturer’s protocol. Based on a previous study, an intestinal lavage extract with 3.8 mg/mL of total protein from unfasted mice has the proteolytic activity equivalent of approximately 180 µg/mL of trypsin-like, 250 µg/mL of chymotrypsin-like, and 96 µg/mL of elastase-like proteases (91).

### *In vitro* reovirus digest assay

Reovirus (~2.2 × 10^9^ particles per reaction) was diluted in VDB containing human trypsin (BioVision, P1228-1000), bovine trypsin (Sigma, T-8003), porcine trypsin (Sigma, T4799), human chymotrypsin (Sigma, SRP6509), or bovine chymotrypsin (Sigma, C3142) at a final concentration of 14 µg/mL. Alternatively, the same number of reovirus particles was added to cathepsin reaction buffer (50 mM sodium acetate pH 5.0, 3 mM DTT, 100 mM NaCl) containing either 4 µM of mouse cathepsin B or 1 µM of mouse cathepsin L, as previously described (35). Reaction mixtures were incubated at 37°C for the indicated timepoints before being processed for Western blot analysis after the addition of 5× protein sample buffer and boiling.

### Immunofluorescence

Reovirus (5%–10% infection by flow cytometry) was diluted in VDB containing mouse intestinal lavage [10%, vol/vol; equivalent to ~18 µg/mL of trypsin-like, ~25 µg/mL of chymotrypsin-like, and ~9.6 µg/mL of elastase-like proteases (91)] or not, followed by incubation at 37°C for 30 min. Reaction mixtures were then neutralized using an equal volume of FBS (Sigma, 20H532)-containing complete media. Meanwhile, confluent monolayers of L929 cells were pre-chilled at 4°C for 1 hour. Treated and untreated viruses were further diluted in serum-free media and added to L929 cells for another hour at 4°C with gentle rocking every 7–10 min. After incubation, virus inocula were removed, and cells were washed twice with serum-free MEM before addition of complete media and incubation at 37°C. At 15–18 hpi, media was removed, and the cells were washed twice with 1× PBS prior to fixation with 4% formaldehyde in PBS for 30 min at room temperature. Formaldehyde was discarded, and the cells were washed twice for 5–10 min each with PBS before blocking and permeabilization for 1 hour at room temperature in 3% NBCS/PBS/0.1% Triton X-100 blocking buffer. Blocking buffer was then replaced with primary antibody (mouse anti-σNS mAb, 1:100 in block buffer, Developmental Studies Hybridoma Bank 2A9) and incubated overnight at 4°C (62). The next day, following removal of the primary antibody, samples were washed thrice with PBS/0.1% Triton X-100 (wash buffer) and incubated with a secondary antibody (goat anti-mouse Alexa Fluor 488, 1:300 in block buffer, Jackson ImmunoResearch 115-545-146) protected from light at room temperature for 1 h. DAPI (0.1 µg/mL; Invitrogen D1306) was added alongside the secondary antibody as a DNA counterstain for fluorescence imaging. The secondary antibody was then removed, and cells were washed three times for 5–10 min each with wash buffer. PBS was then added to each well, and cells were imaged on an EVOS FL Auto Imaging System (Life Technologies).

### Flow cytometry

Cells were washed twice with 1× PBS and detached by incubating in Cellstripper (Corning, 25-056-CI) for 5 min at room temperature. Cells were fixed using 4% formaldehyde in PBS for 30 min at room temperature and then washed twice in flow buffer (3% NBCS/PBS/0.1% Triton X-100). Cells were then resuspended in primary antibody (mouse anti-σNS mAb, 1:500) for overnight incubation at 4°C. Samples were washed twice with flow buffer and incubated with secondary antibody (goat anti-mouse Alexa Fluor 488, 1:1,000), protected from light, for 1 hour at room temperature. Cells were washed two more times and resuspended in PBS. Analysis was performed on the University of Alberta Flow Cytometry Facility’s BD LSR Fortessa (BD Biosciences). A minimum of 10,000 total cells were collected for each sample. Flow cytometry data were analyzed using FlowJo v10 Software (BD Biosciences).

### RT-QPCR

Cells were lysed in TRI Reagent (Millipore Sigma, T9424), and the aqueous phase was separated by chloroform extraction as per the TRI Reagent protocol. Isopropanol was mixed with the aqueous phase, and the RNA was isolated using the GenElute Mammalian Total RNA Miniprep Kit (Millipore Sigma, RTN350) according to the manufacturer’s protocol. RNA was eluted using RNase-free water (Sigma, W4502-1L) and quantified using an ND-1000 NanoDrop spectrophotometer. Using 500 ng RNA per 10 µL reaction, cDNA synthesis was performed with random primers (Fisher, 48190011) and M-MLV reverse transcriptase (ThermoFisher Scientific, 8025013) following the manufacturer’s protocols. After a 1/4 cDNA dilution in mqH_2_O, the cDNA samples were used as templates for RT-qPCR following the SYBR Select (Invitrogen, 4472920) protocol using the indicated reovirus-specific primers and the CFX96 system (BioRAD). All RT-qPCR reactions included a no-reverse transcriptase control. Primers: ReoM3fwd—5′GGTATGAGCCTCCAGGATG3′, ReoM3rev—5′CCTCATCAGCAACCTTCGC3′, T1E1S4_HRMfwd—5′TGACTGGAAACTGCAAGATG3′, T1E1S4_HRMrev—5′GTTCTGTTATACCATCCSG3′, T1E1S4_Bigfwd—5′ATACATGCTAGTTGGACTGC3′, T1E1S4_Bigrev—5′TTCAGTGCCGTCCAATATCT3′.

### Sequence analysis

Amino acid sequences were obtained either directly from the NCBI GenBank or by deducing the translation product of nucleotide sequences using the Expasy Translate tool. For amino acid conservation analysis, reovirus σ3 sequences were aligned using the ClustalOmega program (https://www.ebi.ac.uk/Tools/msa/clustalo/), and conservation scores based on parental Edmonton isolates (T1E1, T2E1, T2E2, T2E3) and 58 full-length σ3 amino acid sequences available on the NCBI GenBank were calculated with the Jalview software. Aggregation propensities (Aggre3D scores) were obtained by analysis of the σ3-μ1C complex 3D structure (PDB: 1JMU) on the Aggrescan3D 2.0 algorithm with default settings (60, 79).

### Statistical analysis

Statistical methods are described in the figure legends for the corresponding experiments. Statistical analyses were performed using GraphPad Prism, version 9.5.1.

## SUPPLEMENTAL MATERIAL

Supplemental tables and figure legends jvi.00828-23-s0001.docx Tables S1 to S3 and legends of Fig S1 to S9.

Fig. S1 jvi.00828-23-s0002.tif Natural reovirus isolates are genetically diverse and distinct from both prototypic strains and other known field isolates.

Fig. S2 jvi.00828-23-s0003.tif Naturally-acquired reoviruses have delayed intracellular uncoating in L929 cells.

Fig. S3 jvi.00828-23-s0004.tif The σ1 protein of wild-type prototypic T3D strain is uniquely sensitive to cleavage by intestinal proteases.

Fig. S4 jvi.00828-23-s0005.tif Lab-adapted versus naturally-derived reoviruses exhibit differential susceptibility to host proteases.

Fig. S5 jvi.00828-23-s0006.tif Lab-adapted versus naturally derived reoviruses exhibit differential susceptibility to host proteases.

Fig. S6 jvi.00828-23-s0007.tif Reovirus adaptation to L929 cells correlates with improved intracellular uncoating.

Fig. S7 jvi.00828-23-s0008.tif Reovirus adaptation to intracellular uncoating occurs through cathepsin B/L-dependent and -independent mechanisms.

Fig. S8 jvi.00828-23-s0009.tif Polymorphisms associated with large plaque-forming phenotype of cell culture-adapted reoviruses represent less than 10% of the parental isolate.

Fig. S9 jvi.00828-23-s0010.tif Genetically distinct σ3 protein sequences were present within the parental T1E1 sewage reovirus isolate.

ASM does not own the copyrights to Supplemental Material that may be linked to, or accessed through, an article. The authors have granted ASM a non-exclusive, world-wide license to publish the Supplemental Material files. Please contact the corresponding author directly for reuse.
