# Supplementary material for: Reovirus genomic diversity confers plasticity for protease utility during adaptation to intracellular uncoating
Source: J Virol. 2023 Sep 25;97(10):e00828-23. doi: 10.1128/jvi.00828-23 (PMC10617468; doi:10.1128/jvi.00828-23)
Supplement: Supplemental tables and figure legends — Tables S1 to S3 and legends of Fig S1 to S9. [file jvi.00828-23-s0001.docx]

**SUPPLEMENTARY TABLES**

**Table S1. Highest identity nucleotide hits from NCBI GenBank for Edmonton wastewater reovirus isolates.**

|  | | **Most closely related isolate** | **Serotype** | **Source** | **Year** | **ID** | **% Identity** | |
| --- | --- | --- | --- | --- | --- | --- | --- | --- |
|  |  |  |  |  |  |  | **nt** | **aa** |
| **T1E1** | **S1** | YNSZ/V207/2016 | 1 | China: Cattle | 2016 | [MW198710.1](https://www.ncbi.nlm.nih.gov/nucleotide/MW198710.1?report=genbank&log$=nuclalign&blast_rank=1&RID=NHG7EK7C016) | 98.2 | 97.4 |
|  | **S2** | MRV-3 chamois 84407 Italy 2009 | 3 | Italy: Chamois | 2009 | [MK408606.1](https://www.ncbi.nlm.nih.gov/nucleotide/MK408606.1?report=genbank&log$=nuclalign&blast_rank=1&RID=NHGHU6JP016) | 97.3 | 100.0 |
|  | **S3** | Osaka2014 | 2 | Japan: Human | 2014 | [LC476923.1](https://www.ncbi.nlm.nih.gov/nucleotide/LC476923.1?report=genbank&log$=nuclalign&blast_rank=1&RID=NHGSP5WH016) | 97.9 | 99.6 |
|  | **S4** | MRV2Tou05 | 2 | France: Human | 2009 | [GU196313.1](https://www.ncbi.nlm.nih.gov/nucleotide/GU196313.1?report=genbank&log$=nuclalign&blast_rank=1&RID=NHGZ4TX0016) | 96.6 | 98.9 |
|  | **M1** | WIV5 | 2 | China: Bat | 2011 | [KT444545.1](https://www.ncbi.nlm.nih.gov/nucleotide/KT444545.1?report=genbank&log$=nuclalign&blast_rank=1&RID=NHH5XUHA016) | 96.7 | 97.9 |
|  | **M2** | SI-MRV04 | 1 | Slovenia: Bat | 2009 | [MG457102.1](https://www.ncbi.nlm.nih.gov/nucleotide/MG457102.1?report=genbank&log$=nuclalign&blast_rank=1&RID=NHHNJHD501N) | 97.6 | 99.6 |
|  | **M3** | MRV2Tou05 | 2 | France: Human | 2009 | [GU196314.1](https://www.ncbi.nlm.nih.gov/nucleotide/GU196314.1?report=genbank&log$=nuclalign&blast_rank=1&RID=NHJFFUY9013) | 97.8 | 98.8 |
|  | **L1** | MRV2Tou05 | 2 | France: Human | 2009 | [GU196306.1](https://www.ncbi.nlm.nih.gov/nucleotide/GU196306.1?report=genbank&log$=nucltop&blast_rank=1&RID=NHJFFUY9013) | 97.7 | 99.3 |
|  | **L2** | MRV2Tou05 | 2 | France: Human | 2009 | [GU196307.1](https://www.ncbi.nlm.nih.gov/nucleotide/GU196307.1?report=genbank&log$=nucltop&blast_rank=1&RID=NHJFFUY9013) | 98.0 | 99.5 |
|  | **L3** | MRV2Tou05 | 2 | France: Human | 2009 | [GU196308.1](https://www.ncbi.nlm.nih.gov/nucleotide/GU196308.1?report=genbank&log$=nucltop&blast_rank=1&RID=NHJFFUY9013) | 97.6 | 99.5 |
| **T2E1** | **S1** | T2W | 2 | Canada: Lab | 2006 | [DQ220017.1](https://www.ncbi.nlm.nih.gov/nucleotide/DQ220017.1?report=genbank&log$=nuclalign&blast_rank=1&RID=NHG7EK7C016) | 95.6 | 93.6 |
|  | **S2** | BtMRV-QAPpC66 | 2 | China: Bat | 2016 | [OP169470.1](https://www.ncbi.nlm.nih.gov/nucleotide/OP169470.1?report=genbank&log$=nuclalign&blast_rank=1&RID=NHGHU6JP016) | 91.3 | 98.3 |
|  | **S3** | FS-03 | 3 | USA: Pig | 2014 | [KM820762.1](https://www.ncbi.nlm.nih.gov/nucleotide/KM820762.1?report=genbank&log$=nuclalign&blast_rank=1&RID=NHGSP5WH016) | 96.9 | 99.2 |
|  | **S4** | R124 | 3 | Netherlands: Lab | 2012 | [GU991668.1](https://www.ncbi.nlm.nih.gov/nucleotide/GU991668.1?report=genbank&log$=nuclalign&blast_rank=1&RID=NHGZ4TX0016) | 94.2 | 97.8 |
|  | **M1** | T1L | 1 | USA: Lab | 2002 | [AF461682.1](https://www.ncbi.nlm.nih.gov/nucleotide/AF461682.1?report=genbank&log$=nuclalign&blast_rank=1&RID=NHH5XUHA016) | 97.5 | 97.7 |
|  | **M2** | T1L | 1 | USA: Lab | 2002 | [AF490617.1](https://www.ncbi.nlm.nih.gov/nucleotide/AF490617.1?report=genbank&log$=nuclalign&blast_rank=1&RID=NHHNJHD501N) | 95.5 | 99.4 |
|  | **M3** | MRV1/40/Bat/Kansas USA/2018 | 1 | USA: Bat | 2018 | [OP057406.1](https://www.ncbi.nlm.nih.gov/nucleotide/OP057406.1?report=genbank&log$=nucltop&blast_rank=1&RID=NHJFFUY9013) | 97.1 | 97.8 |
|  | **L1** | R124 | 3 | Netherlands: Lab | 2012 | [GU991659.1](https://www.ncbi.nlm.nih.gov/nucleotide/GU991659.1?report=genbank&log$=nucltop&blast_rank=1&RID=NHJFFUY9013) | 94.5 | 98.0 |
|  | **L2** | T3D-S | 3 | Canada: Lab | 2002 | [KP208805.1](https://www.ncbi.nlm.nih.gov/nucleotide/KP208805.1?report=genbank&log$=nucltop&blast_rank=1&RID=NHJFFUY9013) | 84.8 | 96.2 |
|  | **L3** | T3A | 3 | Canada: Lab | 2010 | [GU589579.1](https://www.ncbi.nlm.nih.gov/nucleotide/GU589579.1?report=genbank&log$=nucltop&blast_rank=1&RID=NHJFFUY9013) | 96.7 | 99.5 |
| **T2E2** | **S1** | 17-EF40 | 2 | USA: Bat | 2017 | [MW718868.1](https://www.ncbi.nlm.nih.gov/nucleotide/MW718868.1?report=genbank&log$=nuclalign&blast_rank=1&RID=NHG7EK7C016) | 82.2 | 89.1 |
|  | **S2** | 19-LN21 | 2 | USA: Bat | 2019 | [MT498609.1](https://www.ncbi.nlm.nih.gov/nucleotide/MT498609.1?report=genbank&log$=nuclalign&blast_rank=1&RID=NHGHU6JP016) | 90.2 | 99.5 |
|  | **S3** | SD-14 | 3 | China: Mink | 2014 | [KT224512.1](https://www.ncbi.nlm.nih.gov/nucleotide/KT224512.1?report=genbank&log$=nuclalign&blast_rank=1&RID=NHGSP5WH016) | 99.6 | 99.5 |
|  | **S4** | SD-14 | 3 | China: Mink | 2014 | [KT224513.1](https://www.ncbi.nlm.nih.gov/nucleotide/KT224513.1?report=genbank&log$=nuclalign&blast_rank=1&RID=NHGZ4TX0016) | 98.9 | 99.7 |
|  | **M1** | 19-LN21 | 2 | USA: Bat | 2019 | [MT498605.1](https://www.ncbi.nlm.nih.gov/nucleotide/MT498605.1?report=genbank&log$=nuclalign&blast_rank=1&RID=NHH5XUHA016) | 92.8 | 97.8 |
|  | **M2** | 729 | 2 | Austria: Pig | 1998 | [JN799424.1](https://www.ncbi.nlm.nih.gov/nucleotide/JN799424.1?report=genbank&log$=nuclalign&blast_rank=1&RID=NHHNJHD501N) | 96.7 | 99.4 |
|  | **M3** | 19-LN21 | 2 | USA: Bat | 2019 | [MT498607.1](https://www.ncbi.nlm.nih.gov/nucleotide/MT498607.1?report=genbank&log$=nucltop&blast_rank=1&RID=NHJFFUY9013) | 88.2 | 94.6 |
|  | **L1** | 19-LN21 | 2 | USA: Bat | 2019 | [MT498602.1](https://www.ncbi.nlm.nih.gov/nucleotide/MT498602.1?report=genbank&log$=nucltop&blast_rank=1&RID=NHJFFUY9013) | 95.5 | 99.0 |
|  | **L2** | 19-LN21 | 2 | USA: Bat | 2019 | [MT498603.1](https://www.ncbi.nlm.nih.gov/nucleotide/MT498603.1?report=genbank&log$=nucltop&blast_rank=1&RID=NHJFFUY9013) | 86.1 | 94.3 |
|  | **L3** | 19-LN21 | 2 | USA: Bat | 2019 | [MT498604.1](https://www.ncbi.nlm.nih.gov/nucleotide/MT498604.1?report=genbank&log$=nucltop&blast_rank=1&RID=NHJFFUY9013) | 82.9 | 98.6 |
| **T2E3** | **S1** | 17-EF40 | 2 | USA: Bat | 2017 | [MW718868.1](https://www.ncbi.nlm.nih.gov/nucleotide/MW718868.1?report=genbank&log$=nuclalign&blast_rank=1&RID=NHG7EK7C016) | 82.2 | 89.6 |
|  | **S2** | SD-14 | 3 | China: Mink | 2014 | [KT224511.1](https://www.ncbi.nlm.nih.gov/nucleotide/KT224511.1?report=genbank&log$=nuclalign&blast_rank=1&RID=NHGHU6JP016) | 99.6 | 99.7 |
|  | **S3** | SD-14 | 3 | China: Mink | 2014 | [KT224512.1](https://www.ncbi.nlm.nih.gov/nucleotide/KT224512.1?report=genbank&log$=nuclalign&blast_rank=1&RID=NHGSP5WH016) | 99.3 | 99.6 |
|  | **S4** | OV204 | 2 | USA: Deer | 2018 | [MK092973.1](https://www.ncbi.nlm.nih.gov/nucleotide/MK092973.1?report=genbank&log$=nuclalign&blast_rank=1&RID=NHGZ4TX0016) | 99.7 | 99.7 |
|  | **M1** | Osaka2005 | 2 | Japan: Human | 2005 | [LC476908.1](https://www.ncbi.nlm.nih.gov/nucleotide/LC476908.1?report=genbank&log$=nuclalign&blast_rank=1&RID=NHH5XUHA016) | 98.9 | 99.6 |
|  | **M2** | 729 | 2 | Austria: Pig | 1998 | [JN799424.1](https://www.ncbi.nlm.nih.gov/nucleotide/JN799424.1?report=genbank&log$=nuclalign&blast_rank=1&RID=NHHNJHD501N) | 96.8 | 99.7 |
|  | **M3** | WIV3 | 2 | China: Bat | 2011 | [KT444577.1](https://www.ncbi.nlm.nih.gov/nucleotide/KT444577.1?report=genbank&log$=nucltop&blast_rank=1&RID=NHJFFUY9013) | 99.5 | 99.9 |
|  | **L1** | 19-LN21 | 2 | USA: Bat | 2019 | [MT498602.1](https://www.ncbi.nlm.nih.gov/nucleotide/MT498602.1?report=genbank&log$=nucltop&blast_rank=1&RID=NHJFFUY9013) | 96.1 | 99.4 |
|  | **L2** | SD-14 | 3 | China: Mink | 2014 | [KT224505.1](https://www.ncbi.nlm.nih.gov/nucleotide/KT224505.1?report=genbank&log$=nucltop&blast_rank=1&RID=NHJFFUY9013) | 98.7 | 99.4 |
|  | **L3** | OV204 | 2 | USA: Deer | 2018 | [MK092966.1](https://www.ncbi.nlm.nih.gov/nucleotide/MK092966.1?report=genbank&log$=nucltop&blast_rank=1&RID=NHJFFUY9013) | 99.3 | 99.8 |

**Table S2. Amino acid polymorphisms between T1E1 progeny variants.**

| **Plaque size rel. to T3D^PL^** | | | .07 | .08 | .09 | .12 | .15 | .21 | .22 | .22 | .22 | .26 | .33 | .37 | .44 | .44 | .46 | .47 | .49 | .53 |  | |
| --- | --- | --- | --- | --- | --- | --- | --- | --- | --- | --- | --- | --- | --- | --- | --- | --- | --- | --- | --- | --- | --- | --- |
| **Protein/gene** | **Position** | **T1E1** | **v1** | **v2** | **v3** | **v4** | **v5** | **v6** | **v7** | **v8** | **v9** | **v10** | **v11** | **v12** | **v15** | **v16** | **v17** | **v18** | **v19** | **v20** | **T1L** | **T3D^PL^** |
| **σ1/S1** | 52 | F |  | F | L | F | | | | | | |  |  | F | | L | |  |  | L | G |
|  | 78 | V |  | V | M | V | | | | | | |  | V | | | M | V |  | V | V | Q |
|  | 108 | I |  | V | | I | | | | | | |  | I | | | V | |  | I | V | R |
|  | 152 | A | | | T | A | | | | | | | | | | | T | |  | A | T | |
|  | 157 | N | | | S | N | | | | | | | | | | | S | |  | N | G | A |
|  | 159 | S | | | | | | | | | | | | | | | | N |  | S | N | E |
|  | 174 | N | | | T | N | | | | | | | | | | | T | |  | N | N | G |
|  | 200 | K | | | N | K | | | | | | | | | | | N | |  | K | N | A |
|  | 230 | V | | | | | | | | | | | | | | | | A |  | V | V | N |
|  | 249 | N | | | S | N | | | | | | | | | | | S | |  | N | S | T |
|  | 353 | N | | K | N | | | | | | | | | | | | | |  | N | N | A |
| **σ2/S2** | 203 | W | | | | | |  | W | | | | | |  | W | |  |  | C | W | |
| **σNS/S3** | No differences | | | | | | | | | | | | | | | | | | | |  | |
| **μ1/M2** | No differences | | | | | | | | | | | | | | | | | | | |  | |
| **μ2/M1** | 239 | K | | R | K | |  | K | | | | | | | | | | | | | K | |
|  | 245 | I | | V | I | |  | I | | | | | | | | | | | | | I | |
|  | 435 | K | | Q | K | |  | K | | | | | | | | | |  | K | | Q | |
|  | 510 | I | | M | I | |  | I | | | | | | | | | |  | I | | M | |
|  | 513 | K | | Q | K | |  | K | | | | | | | | | |  | K | | K | |
|  | 565 | G | | R | G | |  | G |  | G | | | | | | | |  | G | | G | |
|  | 608 | D | | E | D | |  | D |  | D | | | | | | | |  | D | | E | |
| **μNS/M3** | 234 | D | E | | | | |  | E | | | | D | E |  | E |  | E |  | E | E | |
|  | 310 | I | | | | | |  | I | | | | V | I | | | | | | | V | |
|  | 664 |  | T | | | | |  | T | | | | I | T | |  |  | T |  |  | T | |
| **λ1/L3** | 639 | T | | | | | | A | T | | | | | | | | | | | | T | |
| **λ2/L2** | 662-663 | v16 has "LTA" insertion between residues 662-663 | | | | | | | | | | | | | | | | | | |  |  |
|  | 779 | S | | | | | |  | S | | | | | |  | S | | |  | P | S | |
|  | 838 | E | | | | | |  | E | | | | | | A | E | |  |  | E | E | |
|  | 878 | I | L | I | | | |  | I | | | | | | | | |  |  | I | I | |
|  | 1000 | T | | | | A | T | | | | A | T | | A | T | | |  |  | T | T | |
|  | 1080 | T | | | A | T | | | | | | | | | | | |  |  | T | T | S |
| **λ3/L1** | 392 | S | | | | | | | | | | | | |  | S | |  |  | G | S | |
|  | 924 | C | | | | | | | | | | | | | | | | |  | G | C | |
|  | 934 | R | | | | | | L | R | | | | | | | | | |  | R | R | |
|  | 1166 | D | | | | | |  | D | | | | | |  | D | | G |  |  | D | |

| Sequence unavailable |
| --- |
| Amino acid differences |

**Table S3. GenBank Accession Numbers for naturally-derived and T1E1 progeny viruses sequences.**

| Virus | Gene | Accession Number | Virus | Gene | Accession Number | Virus | Gene | Accession Number | Virus | Gene | Accession Number |
| --- | --- | --- | --- | --- | --- | --- | --- | --- | --- | --- | --- |
| T1E1 | S1 | OQ924304 | **T1E1v2** | M1 | OR074569 | **T1E1v8** | M1 | OR074623 | **T1E1v15** | L2-2 | OR074677 |
|  | S2 | OQ924305 |  | M2 | OR074570 |  | M2 | OR074624 |  | L2-3 | OR074678 |
|  | S3 | OQ924306 |  | M3 | OR074571 |  | M3 | OR074625 |  | L3 | OR074679 |
|  | S4 | OQ924307 |  | L1 | OR074572 |  | L1 | OR074626 | **T1E1v16** | S1 | OR074680 |
|  | M1 | OQ924308 |  | L2 | OR074573 |  | L2 | OR074627 |  | S2 | OR074681 |
|  | M2 | OQ924309 |  | L3 | OR074574 |  | L3 | OR074628 |  | S3 | OR074682 |
|  | M3 | OQ924310 | **T1E1v3** | S1 | OR074575 | **T1E1v9** | S1 | OR074629 |  | S4 | OR074683 |
|  | L1 | OQ924311 |  | S2 | OR074576 |  | S2 | OR074630 |  | M1 | OR074684 |
|  | L2 | OQ924312 |  | S3 | OR074577 |  | S3 | OR074631 |  | M2 | OR074685 |
|  | L3 | OQ924313 |  | S4 | OR074578 |  | S4 | OR074632 |  | M3 | OR074686 |
| T2E1 | S1 | OR074525 |  | M1 | OR074579 |  | M1 | OR074633 |  | L1-1 | OR074687 |
|  | S2 | OR074526 |  | M2 | OR074580 |  | M2 | OR074634 |  | L1-2 | OR571910 |
|  | S3 | OR074527 |  | M3 | OR074581 |  | M3 | OR074635 |  | L2 | OR074688 |
|  | S4 | OR074528 |  | L1 | OR074582 |  | L1 | OR074636 |  | L3 | OR074689 |
|  | M1 | OR074529 |  | L2 | OR074583 |  | L2 | OR074637 | **T1E1v17** | S1 | OR074690 |
|  | M2 | OR074530 |  | L3 | OR074584 |  | L3 | OR074638 |  | S2 | OR074691 |
|  | M3 | OR074531 | **T1E1v4** | S1 | OR074585 | **T1E1v10** | S1 | OR074639 |  | S3 | OR074692 |
|  | L1 | OR074532 |  | S2 | OR074586 |  | S2 | OR074640 |  | S4 | OR074693 |
|  | L2 | OR074533 |  | S3 | OR074587 |  | S3 | OR074641 |  | M1 | OR074694 |
|  | L3 | OR074534 |  | S4 | OR074588 |  | S4 | OR074642 |  | M2 | OR074695 |
| T2E2 | S1 | OR074535 |  | M1 | OR074589 |  | M1 | OR074643 |  | M3 | OR074696 |
|  | S2 | OR074536 |  | M2 | OR074590 |  | M2 | OR074644 |  | L1 | OR074697 |
|  | S3 | OR074537 |  | M3 | OR074591 |  | M3 | OR074645 |  | L2 | OR074698 |
|  | S4 | OR074538 |  | L1 | OR074592 |  | L1 | OR074646 |  | L3 | OR074699 |
|  | M1 | OR074539 |  | L2 | OR074593 |  | L2 | OR074647 | **T1E1v18** | S1 | OR074700 |
|  | M2 | OR074540 |  | L3 | OR074594 |  | L3 | OR074648 |  | S3 | OR074701 |
|  | M3 | OR074541 | **T1E1v5** | S1 | OR074595 | **T1E1v11** | S1 | OR074649 |  | S4 | OR074702 |
|  | L1 | OR074542 |  | S2 | OR074596 |  | S2 | OR074650 |  | M1 | OR074703 |
|  | L2 | OR074543 |  | S3 | OR074597 |  | S3 | OR074651 |  | M2 | OR074704 |
|  | L3 | OR074544 |  | S4 | OR074598 |  | S4 | OR074652 |  | M3 | OR074705 |
| T2E3 | S1 | OR074545 |  | M3 | OR074599 |  | M1 | OR074653 |  | L1 | OR074706 |
|  | S2 | OR074546 |  | L1 | OR074600 |  | M2 | OR074654 |  | L2 | OR074707 |
|  | S3 | OR074547 |  | L2 | OR074601 |  | M3 | OR074655 |  | L3 | OR074708 |
|  | S4 | OR074548 |  | L3 | OR074602 |  | L1 | OR074656 | **T1E1v19** | S2 | OR074709 |
|  | M1 | OR074549 | **T1E1v6** | S1 | OR074603 |  | L2 | OR074657 |  | S3 | OR074710 |
|  | M2 | OR074550 |  | S4 | OR074604 |  | L3 | OR074658 |  | S4 | OR074711 |
|  | M3 | OR074551 |  | M1 | OR074605 | **T1E1v12** | S1 | OR074659 |  | M1 | OR074712 |
|  | L1 | OR074552 |  | L1 | OR074606 |  | S2 | OR074660 |  | M2 | OR074713 |
|  | L2 | OR074553 |  | L2-1 | OR074607 |  | S3 | OR074661 |  | M3 | OR074714 |
|  | L3 | OR074554 |  | L2-2 | OR487159 |  | S4 | OR074662 |  | L2 | OR074715 |
| T1E1v1 | S1 | OR074555 |  | L3 | OR074608 |  | M1 | OR074663 |  | L3 | OR074716 |
|  | S2 | OR074556 | **T1E1v7** | S1 | OR074609 |  | M2 | OR074664 | **T1E1v20** | S1 | OR074717 |
|  | S3 | OR074557 |  | S2 | OR074610 |  | M3 | OR074665 |  | S2 | OR074718 |
|  | S4 | OR074558 |  | S3 | OR074611 |  | L1 | OR074666 |  | S3 | OR074719 |
|  | M1 | OR074559 |  | S4 | OR074612 |  | L2 | OR074667 |  | S4 | OR074720 |
|  | M2 | OR074560 |  | M1 | OR074613 |  | L3 | OR074668 |  | M1 | OR074721 |
|  | M3 | OR074561 |  | M2 | OR074614 | **T1E1v15** | S1 | OR074669 |  | M2 | OR074722 |
|  | L1 | OR074562 |  | M3 | OR074615 |  | S3 | OR074670 |  | M3 | OR074723 |
|  | L2 | OR074563 |  | L1 | OR074616 |  | S4 | OR074671 |  | L1-1 | OR074724 |
|  | L3 | OR074564 |  | L2 | OR074617 |  | M1 | OR074672 |  | L1-2 | OR074725 |
| T1E1v2 | S1 | OR074565 |  | L3 | OR074618 |  | M2 | OR074673 |  | L1-3 | OR074726 |
|  | S2 | OR074566 | **T1E1v8** | S1 | OR074619 |  | M3 | OR074674 |  | L2 | OR074727 |
|  | S3 | OR074567 |  | S2 | OR074620 |  | L1 | OR074675 |  | L3 | OR074728 |
|  | S4 | OR074568 |  | S3 | OR074621 |  | L2-1 | OR074676 |  |  |  |

**SUPPLEMENTARY FIGURE LEGENDS**

**Fig S1. Natural reovirus isolates are genetically diverse and distinct from both prototypic strains and other known field isolates. (A-B)** Whole-genome sequencing was performed on genomic RNA extracted from purified effluent reovirus particles. **(A)** The M3 gene nucleotide sequence of naturally-acquired isolates (black diamond) and others available on the NCBI GenBank were aligned using ClustalOmega with default settings. Phylogenetic trees were generated using the maximum-likelihood method based on the Tamura-Nei model with 1000 bootstrap replicates on MEGA 11 software(47). The scale bar represents the number of substitutions per site. **(B)** Percent identity matrices comparing the S2, S3, S4, M1, M2, L2, or L3 genes of prototypic lab strains and Edmontonian isolates at the nucleotide (y-axis) and amino acid levels (x-axis) following pairwise comparison analysis on ClustalOmega

**Fig S2. Naturally-acquired reoviruses have delayed intracellular uncoating in L929 cells.**

Similar to Figure 2, L929 cells were synchronously infected with T1L, T3D^PL^, T1E1, T2E1, T2E2, or T2E3 at 4°C for 1 hour. Following incubation, cells were washed twice with serum-free media and placed in **(A)** regular complete growth media or **(B)** complete growth media supplemented with 100 μg/mL cycloheximide at 37°C, representing 0hpi. At the indicated timepoints, cells were harvested using RIPA buffer and processed for immunoblotting using anti-reovirus and anti-β-actin antibodies**.**

**Fig S3. The σ1 protein of wild-type prototypic T3D strain is uniquely sensitive to cleavage by intestinal proteases. (A)** T1L, T3D^PL^, T1E1, T2E1, T2E2, and T2E3 virions were mixed with bovine chymotrypsin (14μg/mL) in VDB before being incubated for 3h at 37°C. At the indicated timepoints, samples were processed for immunoblotting using anti-T1 or -T3 σ1 antibodies. **(B)** Similar to (A), except reoviruses were treated with 10% mouse intestinal lavage in VDB for 30min at 37°C instead and T3D^PL(T249I)^ virus was also included.

**Fig S4. Lab-adapted versus naturally-derived reoviruses exhibit differential susceptibility to host proteases. (A-C)** Equal number of T1L, T3D^PL^, T1E1, T2E1, T2E2, or T2E3 particles (~2.2 x 10^9^) were mixed with recombinant intestinal proteases (14μg/mL) before being incubated at 37°C. At the indicated timepoints, samples were processed for immunoblotting using anti-reovirus antibodies. **(A)** Blots with bovine trypsin. **(B)** Blots with bovine chymotrypsin. **(C)** Blots with porcine trypsin.

**Fig S5. Lab-adapted versus naturally-derived reoviruses exhibit differential susceptibility to host proteases.** Uncoating kinetics curves of percent σ3 degradation and μ1C-to-δ cleavage over time during *in vitro* reovirus digestions with intestinal and lysosomal proteases. The σ3 degradation was measured by comparing σ3 protein band intensities relative to virus alone while μ1C-to-δ conversion was assessed as the proportion of δ relative to δ+μ1C. Error bars represent the standard deviation from at least N= 3 independent experiments.

**Fig S6. Reovirus adaptation to L929 cells correlates with improved intracellular uncoating. (A)** Monolayers of L929 cells were infected with parental T1E1 or culture-adapted progenies and overlaid with agar containing media prior to incubation at 37°C. Cells were fixed at 6 days post-infection prior to blocking and probing using anti-reovirus primary antibodies. Following incubation with AP-conjugated secondary antibodies, cells were processed for BCIP/NBT staining to produce plaques of infected cells. Foci size was quantified using ImageJ and plotted relative to T3D^PL^. Progeny viruses are ordered by increasing plaque size. **(B)** L929 cells were synchronously infected with parental T1E1, T1E1 progenies, or T3D^PL^ at 4°C for 1h. Following incubation, cells were washed twice with serum-free media, harvested using RIPA buffer, and processed for immunoblotting using anti-reovirus and anti-β-actin antibodies**. (C)** Similar to (B), except infected cell lysates were transferred to 37°C after washes and addition of growth media. Cell lysate was collected at multiple timepoints post-infection. The uncoating percentage 50 (UP50) represents the amount of time (in hours) required to obtain 50% μ1C-to-δ cleavage (i.e., proportion of δ relative to δ+μ1) as calculated by the Nonlinear Fit – Variable Slope (4 parameters) analysis function in Prism 9. Error bars represent the standard deviation for at least N= 2 (plaque size) or 3 (binding and UP50) biological replicates. *P<0.05, **P<0.01, ***P<0.001, ****P<0.0001 by one-way ANOVA with Tukey’s multiple comparison test.

**Fig S7. Reovirus adaptation to intracellular uncoating occurs in cathepsins B/L-dependent and -independent mechanisms.** L929 cells were pre-treated with Cathepsin L Inhibitor III (Cat L_i_, 10μM), Ca-074 Me (Cathepsin B inhibitor, Cat B_i_, 1μM), Cat L_i_+B_i_, or DMSO untreated control. Cells were then synchronously infected with T1E1, T1E1v1, T1E1v5, T1E1v12, T1E1v18, or prototypic lab strains at 4°C for 1 hour. Following incubation, cells were washed twice with serum-free media and placed at 37°C in growth media containing the corresponding drug treatment. At the indicated timepoints post-infection, cells were harvested with RIPA buffer and processed for immunoblotting using anti-reovirus and anti-β-actin antibodies. The percent uncoating over time was calculated as the relative σ3 protein band intensities relative to 0hpi or proportion of δ relative to δ+μ1C.

**Fig S8. Polymorphisms associated with large plaque-forming phenotype of cell culture-adapted reoviruses represent less than 10% of the parental isolate.** RNA was extracted from L929 cells infected with either T1E1v3, T1E1v20, or T1E1 and diluted until the levels of viral M3 was equalized as measured by qPCR. Normalized T1E1v3 and T1E1v20 RNA were mixed in different proportions between 0 to 100% and analyzed by high-resolution melt qPCR to generate control melt curves. Primers were designed to flank a region rich in polymorphisms distinguishing T1E1v3 and v20. Melt curve of the parental T1E1 (green) was compared to controls.

**Fig S9. Genetically distinct σ3 protein sequences were present within the parental T1E1 sewage reovirus isolate.** The consensus σ3 amino acid sequences of large- and small-plaque T1E1 progenies (boxed in red) and others available on the NCBI GenBank were aligned using ClustalOmega with default settings. Phylogenetic trees were generated using the maximum-likelihood method based on the Jones-Taylor-Thornton model with 1000 bootstrap replicates on MEGA 11 software(47). Horizontal branch lengths are drawn to scale with scale bars representing the number of substitutions per site. Percent identity matrix comparing the σ3 amino acid sequences of T1E1 progenies to other isolates available online at the amino acid level following pairwise comparison analysis on ClustalOmega. Consensus small-plaque forming σ3 sequence is highlighted in yellow and large-plaque σ3 sequence in orange.
