# Supplementary figures and images for: Reovirus genomic diversity confers plasticity for protease utility during adaptation to intracellular uncoating
Source: J Virol. 2023 Sep 25;97(10):e00828-23. doi: 10.1128/jvi.00828-23 (PMC10617468; doi:10.1128/jvi.00828-23)

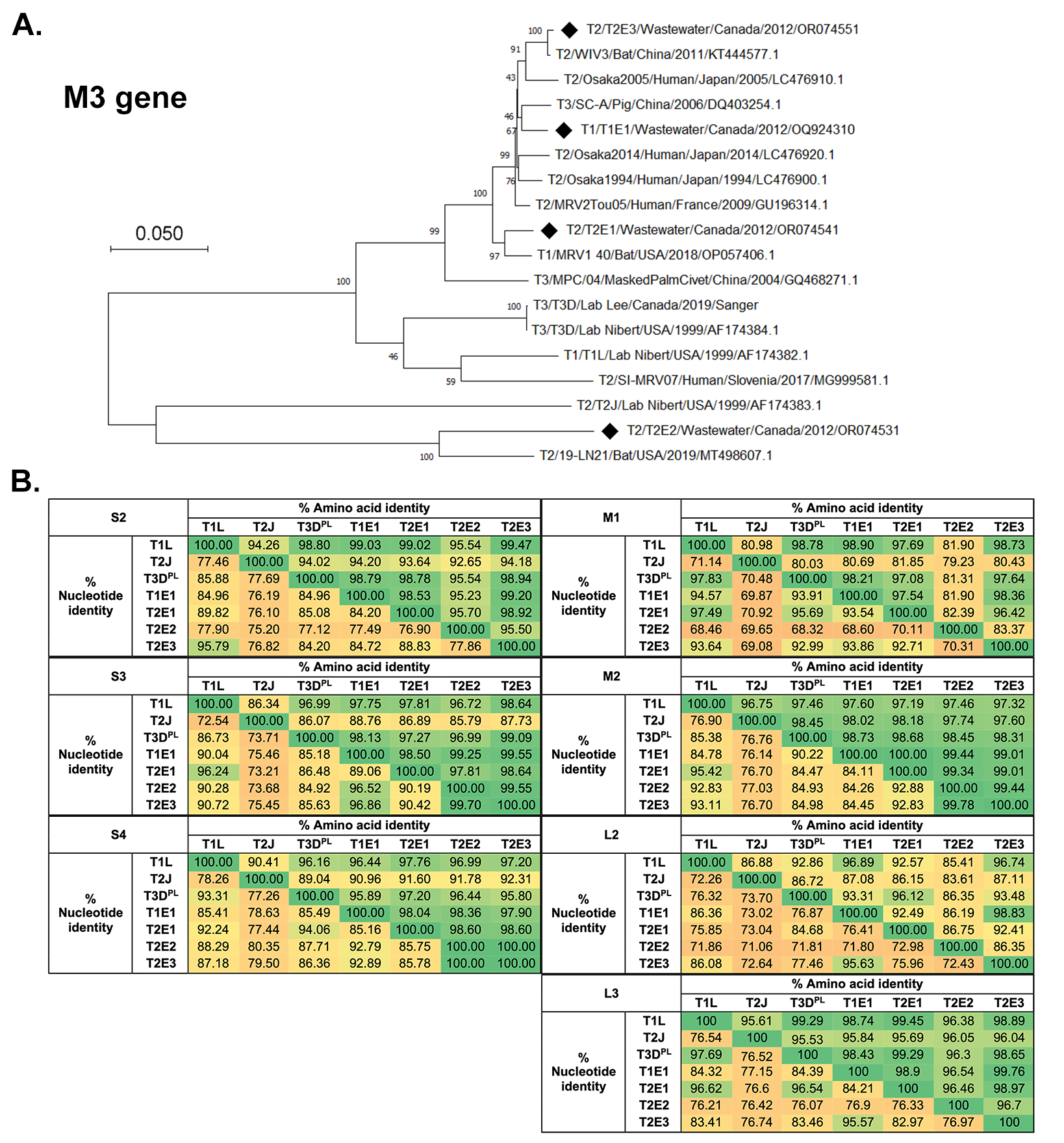

Supplement: Fig. S1 — Natural reovirus isolates are genetically diverse and distinct from both prototypic strains and other known field isolates. [file jvi.00828-23-s0002.tif]

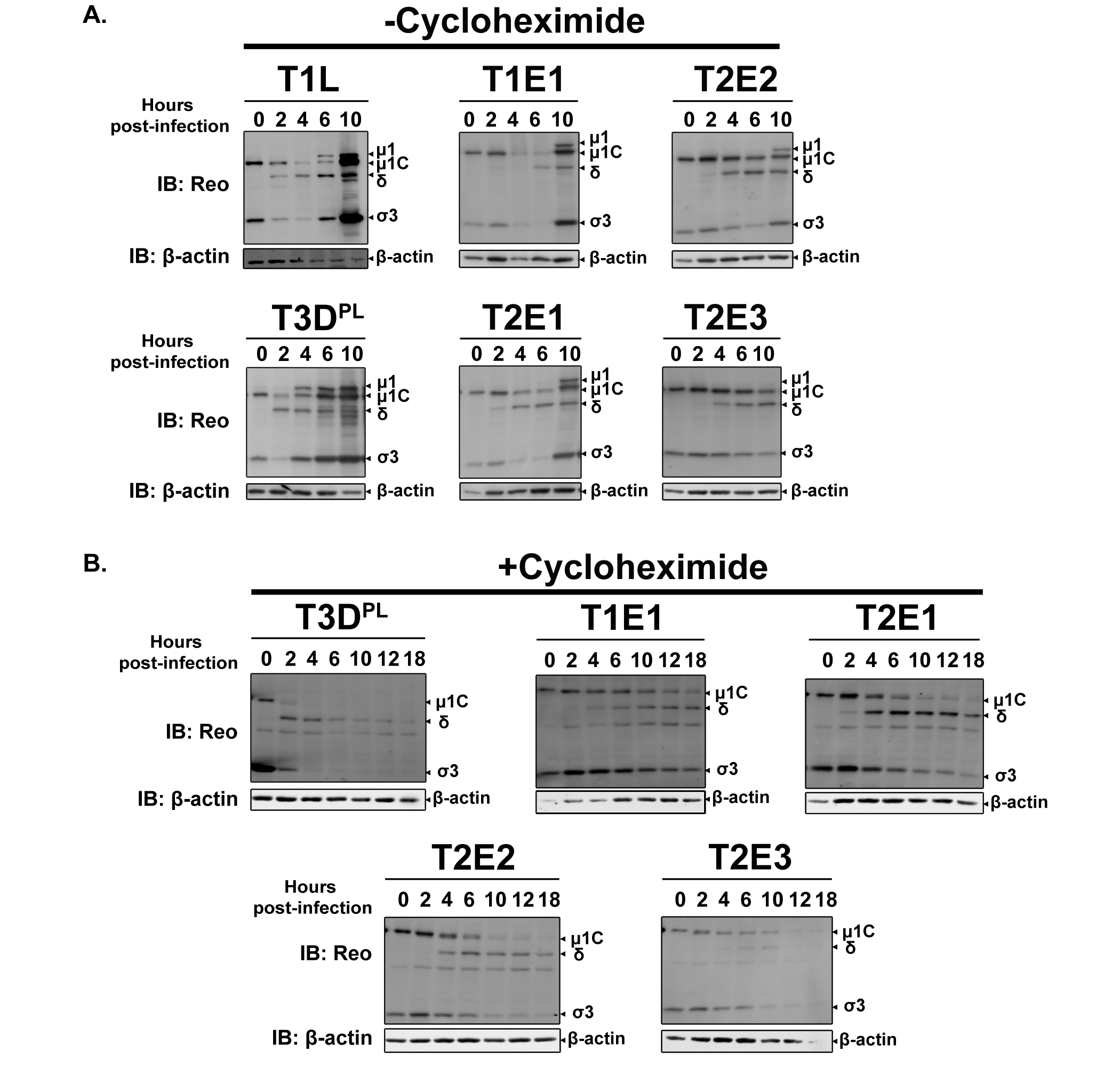

Supplement: Fig. S2 — Naturally-acquired reoviruses have delayed intracellular uncoating in L929 cells. [file jvi.00828-23-s0003.tif]

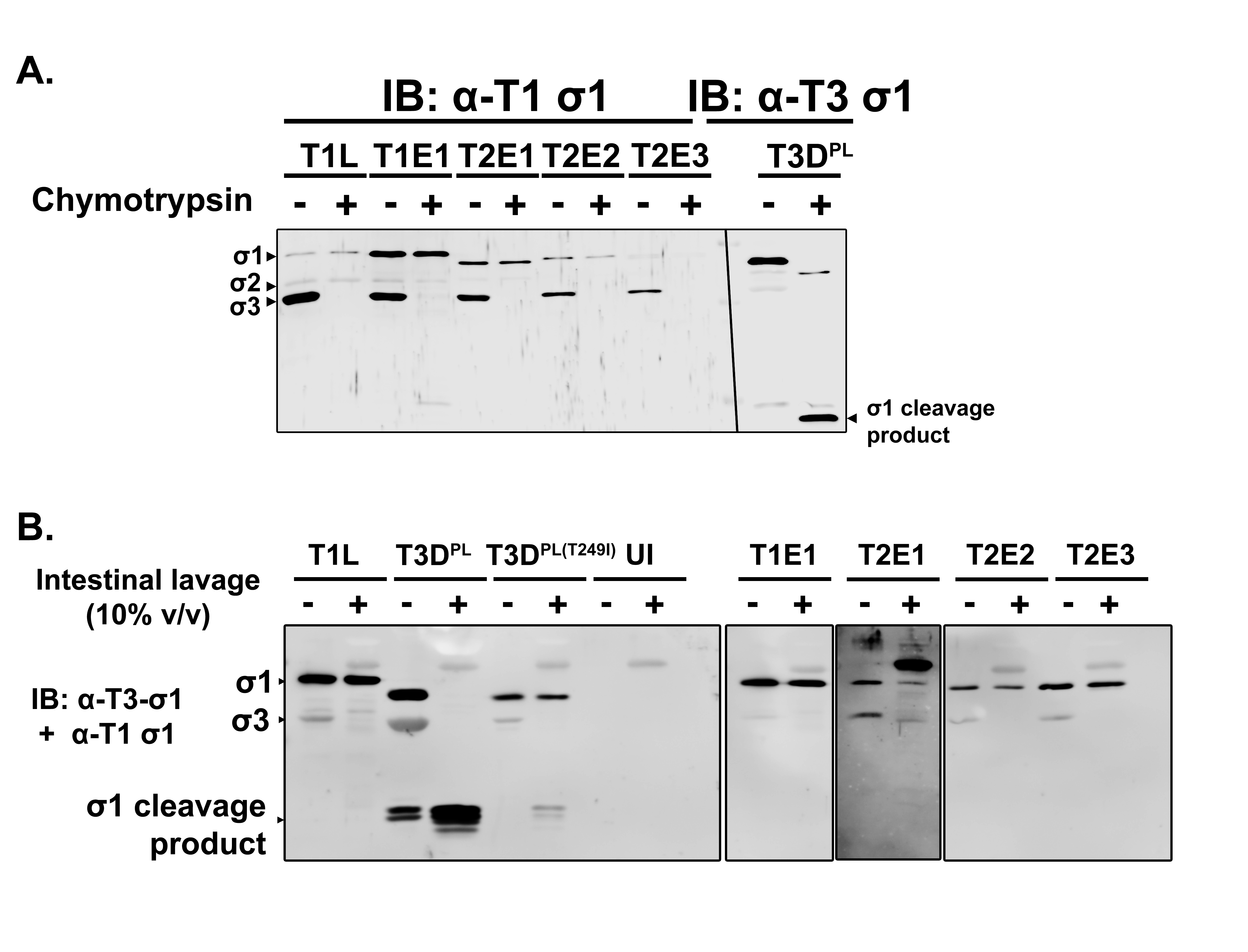

Supplement: Fig. S3 — The σ1 protein of wild-type prototypic T3D strain is uniquely sensitive to cleavage by intestinal proteases. [file jvi.00828-23-s0004.tif]

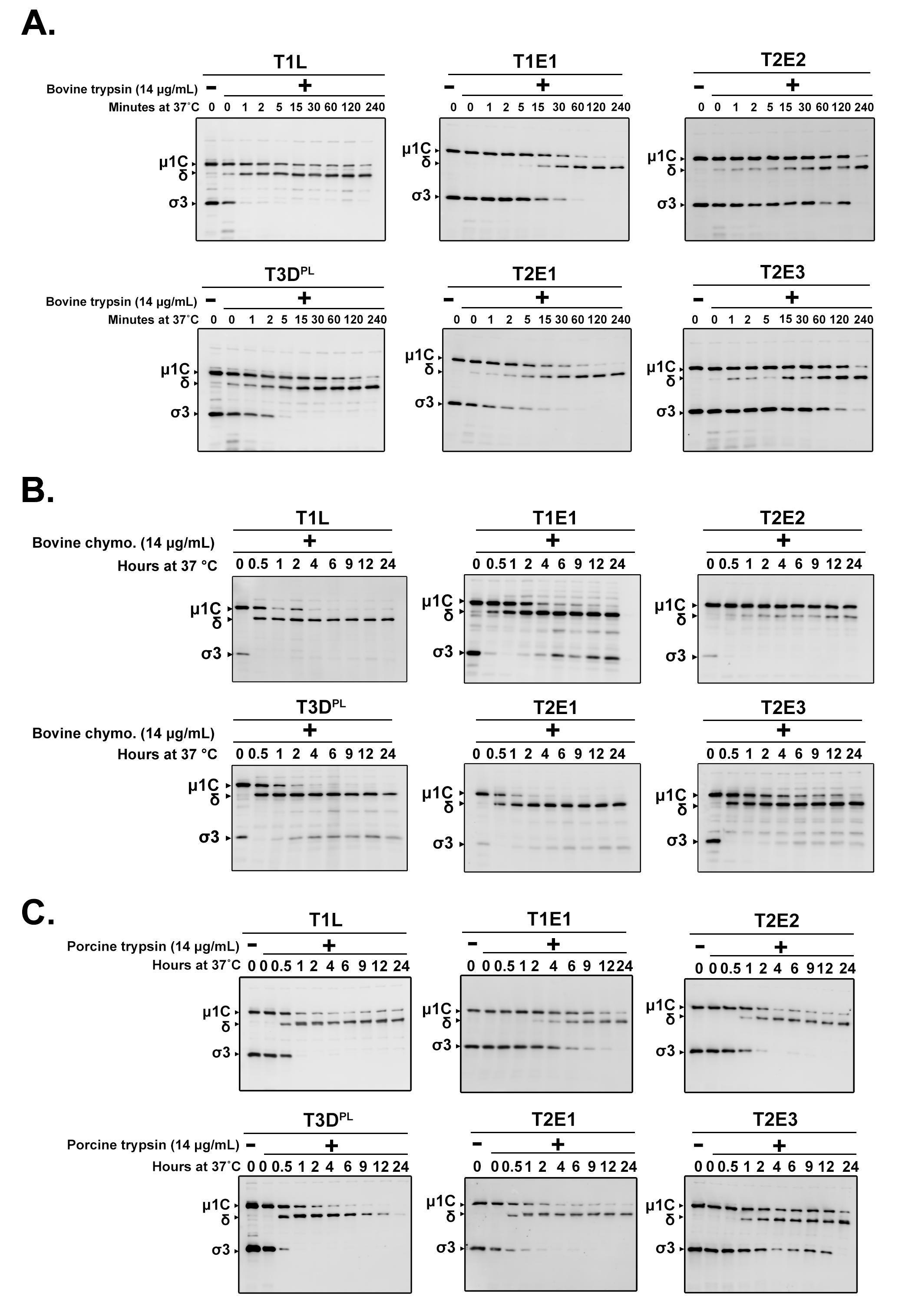

Supplement: Fig. S4 — Lab-adapted versus naturally-derived reoviruses exhibit differential susceptibility to host proteases. [file jvi.00828-23-s0005.tif]

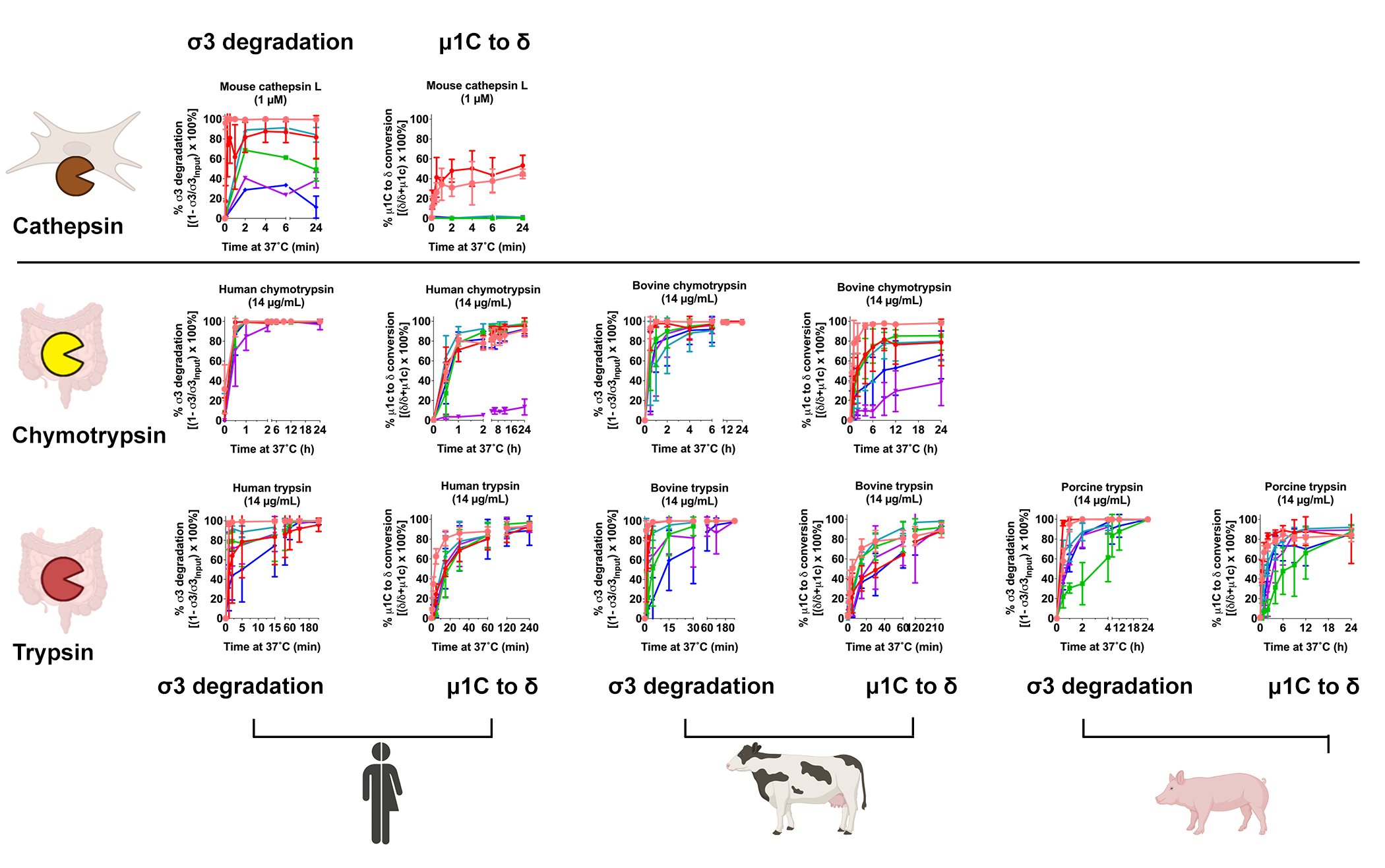

Supplement: Fig. S5 — Lab-adapted versus naturally derived reoviruses exhibit differential susceptibility to host proteases. [file jvi.00828-23-s0006.tif]

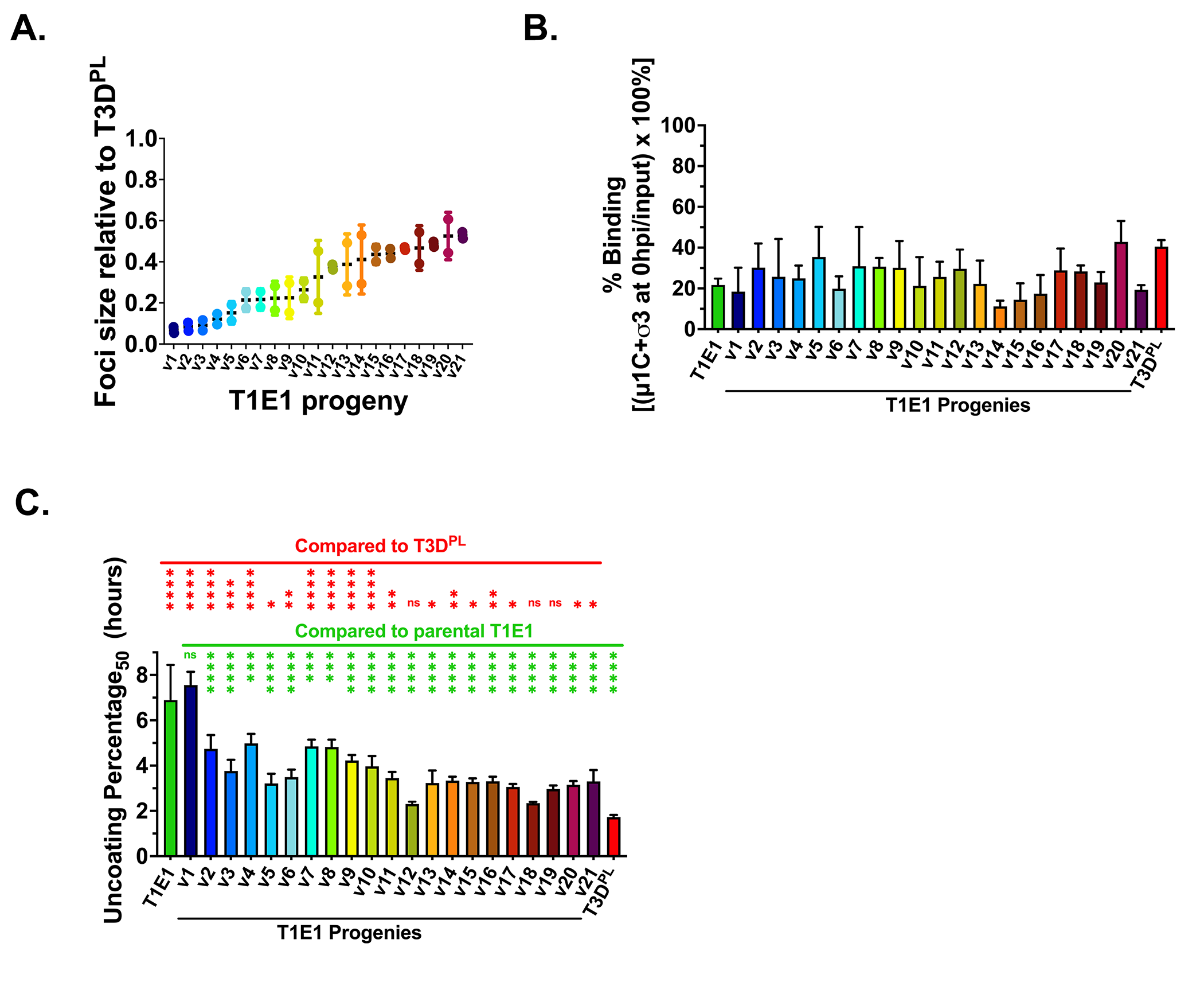

Supplement: Fig. S6 — Reovirus adaptation to L929 cells correlates with improved intracellular uncoating. [file jvi.00828-23-s0007.tif]

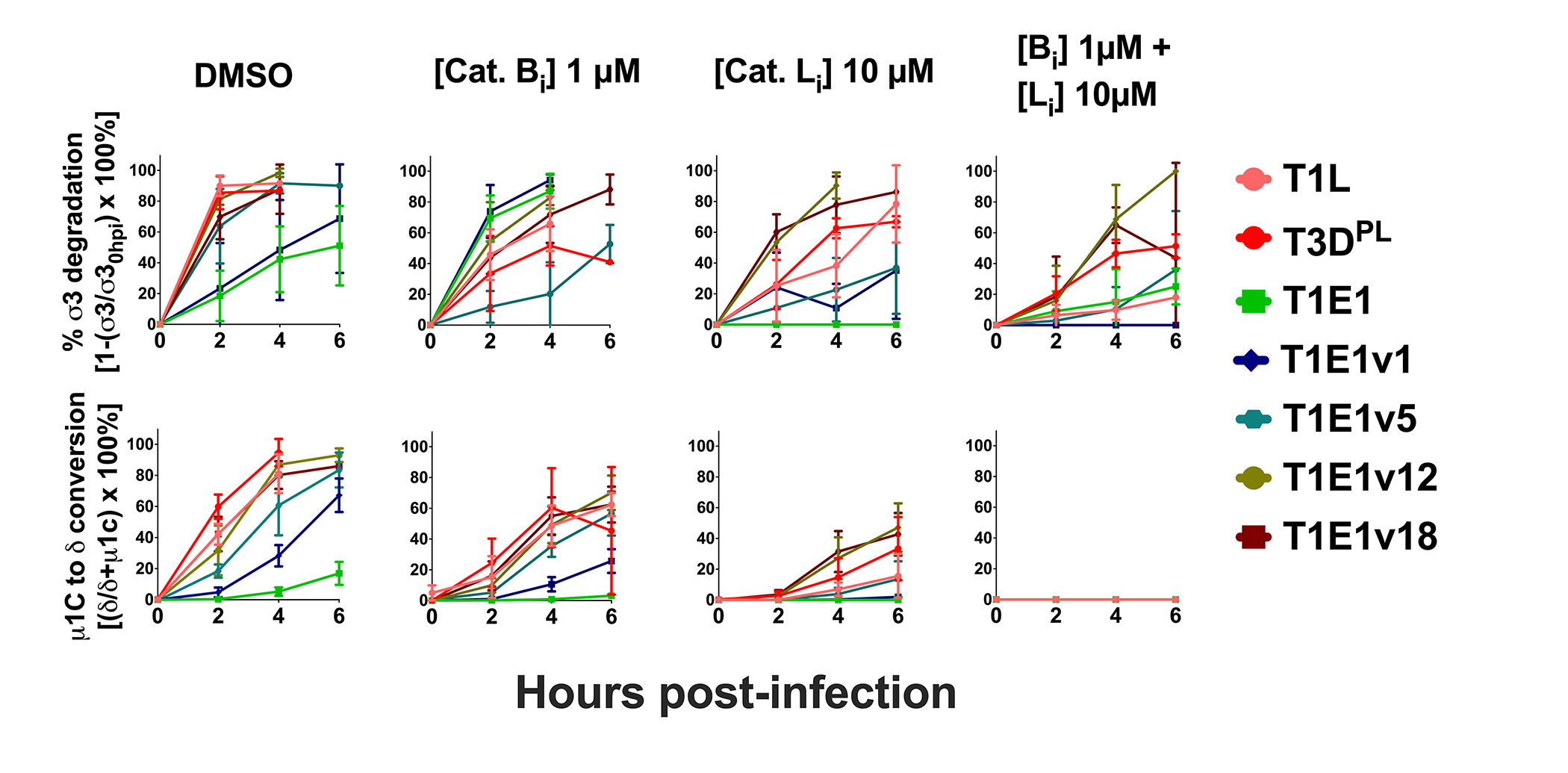

Supplement: Fig. S7 — Reovirus adaptation to intracellular uncoating occurs through cathepsin B/L-dependent and -independent mechanisms. [file jvi.00828-23-s0008.tif]

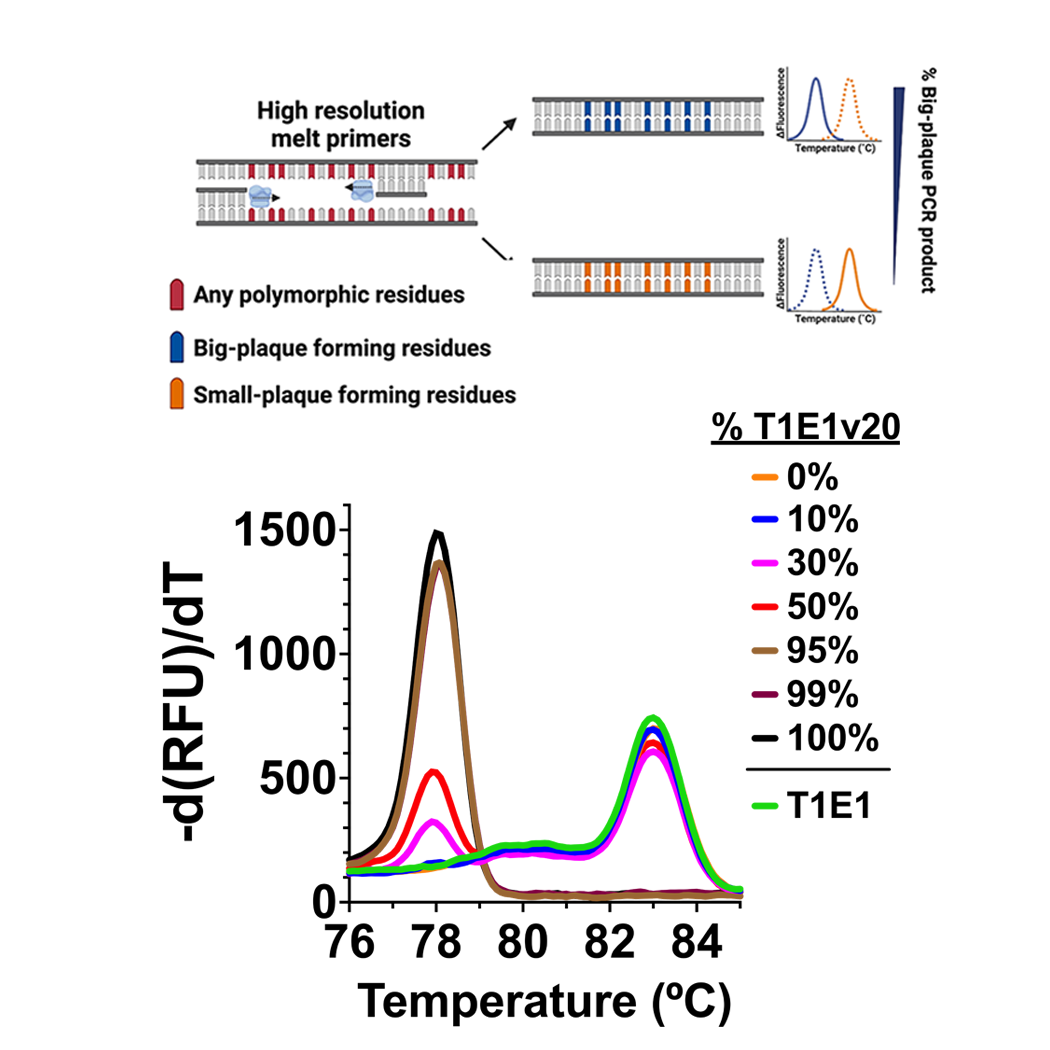

Supplement: Fig. S8 — Polymorphisms associated with large plaque-forming phenotype of cell culture-adapted reoviruses represent less than 10% of the parental isolate. [file jvi.00828-23-s0009.tif]

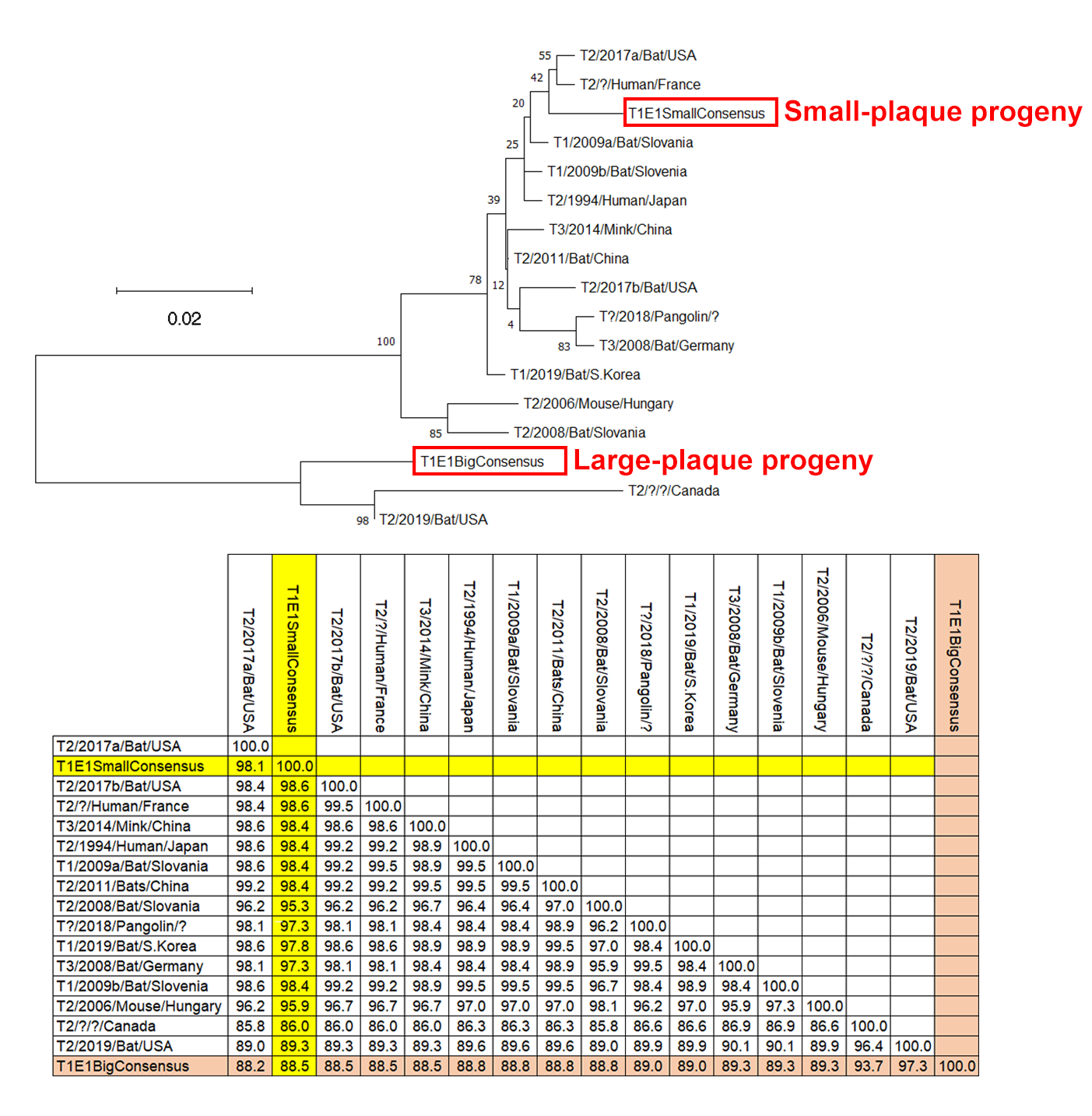

Supplement: Fig. S9 — Genetically distinct σ3 protein sequences were present within the parental T1E1 sewage reovirus isolate. [file jvi.00828-23-s0010.tif]
